# Enhancing and accelerating cell type deconvolution of large-scale spatial transcriptomics slices with dual network model

**DOI:** 10.1093/bioinformatics/btaf419

**Published:** 2025-07-24

**Authors:** Yuhong Zha, Shaoqing Feng, Peng Gao, Quan Zou, Xiaoke Ma

**Affiliations:** School of Computer Science and Technology, Xidian University, Xi’an, Shaanxi 710071, China; Key Laboratory of Smart Human-Computer Interaction and Wearable Technology of Shaanxi Province, Xidian University, Xi’an, Shaanxi 710071, China; Department of Plastic and Reconstructive Surgery, Shanghai Ninth People’s Hospital, Shanghai Jiaotong University, Shanghai 200011, China; Department of Hematology, The First Affiliated Hospital of Xi’an Jiaotong University, Xi’an, Shaanxi 710061, China; Genome Institute, The First Affiliated Hospital of Xi’an Jiaotong University, Xi’an, Shaanxi 710061, China; Institute of Fundamental and Frontier Sciences, University of Electronic Science and Technology of China, Chengdu, Sichuan 611731, China; School of Computer Science and Technology, Xidian University, Xi’an, Shaanxi 710071, China; Key Laboratory of Smart Human-Computer Interaction and Wearable Technology of Shaanxi Province, Xidian University, Xi’an, Shaanxi 710071, China

## Abstract

**Motivation:**

Cell type deconvolution deciphers spatial distribution of mRNA transcripts at single cell level by integrating single-cell RNA sequencing (scRNA-seq) and spatial transcriptomics data to infer mixture of cell types of spots in slices. Current algorithms are criticized for neglecting connection between scRNA-seq and spatial transcriptomics data, as well as time-consuming, hampering their application to large-scale datasets.

**Results:**

In this study, we propose a **j**oint learning nonnegative **m**atrix **f**actorization algorithm for **f**ast cell type **d**econvolution (aka jMF2D), which integrates scRNA-seq and spatial transcriptomics data with network models. To bridge scRNA-seq and spatial transcriptomics data, jMF2D jointly learns cell type similarity network to enhance quality of signatures of cell types, thereby promoting accuracy and efficiency of deconvolution. Experiments demonstrate that jMF2D outperforms state-of-the-art baselines in terms of accuracy by saving about 90% running time on various datasets generated by different platforms. Furthermore, it can also facilitates the identification of spatial domains and bio-marker genes, providing an efficient and effective model for analyzing spatial transcriptomics data.

**Availability and implementation:**

The software is coded using python, and is free available for academic https://github.com/xkmaxidian/jMF2D.

## 1 Introduction

Cells are basic units of tissues and organisms, which execute biological functions via groups of cells with similar activities and behaviors (Ma *et al.* 2020, Zhu *et al.* 2025b). Microarray and the next generation sequencing (RNA-seq), are bulk-based approaches solely measure average expression level of transcripts from various cells, failing to characterize specificity of cells, whereas scRNA-seq technology precisely measures whole-genome wide gene expression at the cell resolution (Chen *et al.* 2017, Dai *et al.* 2025, Dong *et al.* 2025), providing a great opportunity for cell patterns. However, scRNA-seq fails to capture spatial location of cells.

Fortunately, spatial transcriptomics technologies overcome this limitation by simultaneously detecting expression of transcripts and preserving spatial locations of cells, paving the way to investigate spatial distribution of gene expression. On the basis of principles of technologies, current spatial transcriptomics technologies can be roughly divided into two classes, i.e. image-based and sequencing-based. Specifically, the former ones obtain spatial distribution of transcripts with in situ hybridization and fluorescence microscopy, including MERFISH (Moffitt *et al.* 2018), seqFISH (Eng *et al.* 2019), osmFISH (Codeluppi *et al.* 2018), which achieve high resolution by sacrificing genome-scale coverage. In contrast, the latter ones utilize next-generation sequencing to capture transcripts at whole-genome wide, such as 10× Visium (Ståhl *et al.* 2016), and Slide-seq (Stickels *et al.* 2021). But, these approaches are criticized for enlarging coverage of genome by sacrificing resolution of units, i.e. each spot (10–100 mm) may be composed of multiple cell types, making it difficult to capture gene expression at single-cell resolution.

Thus, cell type deconvolution aims to bridges this gap between coverage and resolution by estimating abundance of cell types of each spots, i.e. assigning cell types from scRNA-seq data to spatial locations. Two critical techniques are involved, i.e. how to represent cell types from scRNA-seq data, and how to estimate abundance of cell types for each spot. The first issue can be solved by clustering of scRNA-seq, whereas the second one is much more complicated. Current algorithms estimate abundance of cell types for each spot with various strategies, which are classified into two categories, i.e. direct inference and data fusion methods. The former ones directly infer mixture of cell types for each spot with annotations from scRNA-seq data. For example, Cell2location (Kleshchevnikov *et al.* 2022) uses probabilistic model to estimate abundance of cell types for each spot, whereas RCTD (Cable *et al.* 2022) decomposes cell type mixtures with the supervised learning. SpatialDWLS (Dong and Yuan 2021) deciphers cell type composition of spots with a linear model with the least square strategy, and Stereoscope (Andersson *et al.* 2020) leverages probabilistic model for cell mixtures. SPOTlight (Elosua-Bayes *et al.* 2021) adopts seeded nonnegative matrix factorization (NMF) for cell type deconvolution of spots. These algorithms achieve an excellent performance for cell type deconvolution. However, these algorithms also have limitations for accuracy and efficiency. For example, probabilistic models are not precise for rare and medium size cell types since they require large samples to accurately estimate conditional probability.

The latter ones use additional information for cell type deconvolution, and which are proven to be powerful to enhance performance of algorithms. These algorithms differ greatly on how to select and manipulate these additional information for cell type deconvolution. For example, Redeconve (Zhou *et al.* 2023) utilizes states of cells to facilitate the estimation of abundance of cell types of spots, and DSTG (Song and Su 2021) uses graph convolution networks (GCN) to exploit indirected relations among cells for cell mixture. CARD (Ma and Zhou 2022) makes use of cell-type composition information across locations to improve accuracy of deconvolution, and STRIDE (Sun *et al.* 2022) leverages topic profiles of scRNA-seq data to balance specificity and sensitivity of information. DestVI (Lopez *et al.* 2022) utilizes the continuous variation of transcriptome within cells of the same type, which facilitates optimization of mixture of cell types. MACD (Huang *et al.* 2024) adopts adversarial learning, whereas eMCI (Hong *et al.* 2024) leverages spot-cell correlations for cell type deconvolution.stGNN (Zhu *et al.* 2025a) leverages spatial information to enhance cell type deconvolution. In comparison of direct inference algorithms, these approaches significantly improve accuracy of cell type deconvolution and extend application of spatial transcriptomics (Li *et al.* 2022), which is also one of the major motivations of this study.

**Figure 1.**
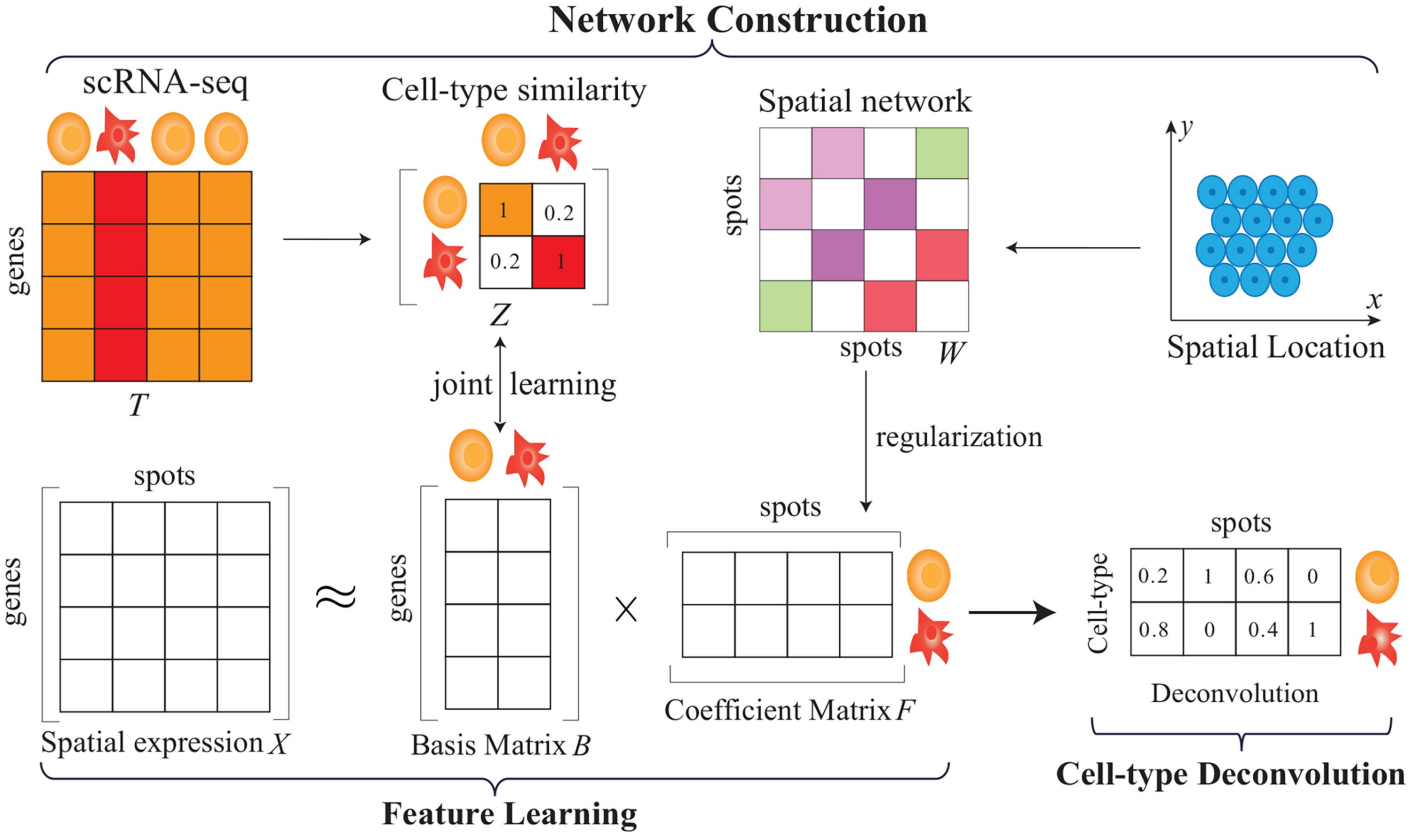
Overview of the jMF2D algorithm for cell type deconvolution, which consists of three major components, i.e. network construction, feature learning, and cell type deconvolution. On the network construction issue, jMF2D automatically learns cell type network *Z* with self-representation learning from signature profiles of cell types, i.e. the average of expression profiles of cells within cell types *Y*. And, it constructs the spot spatial network with KNN *W*, where edge weights are in reverse proportional to Eculidean distance of spatial locations of spots. The feature learning procedure uses NMF to approximately decompose expression profiles of spots *X* into the product of features of cell types *B* (basis matrix) and spots *F*, where the local topological structure of networks of cell types and spots is preserved with regularization. Abundance of cell types in each spot is estimated with deconvolution procedure. Notice that jMF2D integrates all these procedure with a joint learning framework.

Even though great efforts are devoted to cell type deconvolution, there are still many unsolved problems. First, current algorithms first obtain cell types from scRNA-seq data, and then decipher abundance of cell types of spots, which implicitly assume that scRNA-seq and spatial transcriptomics data are independent. Second, available algorithms independently perform cell type convolution for each spot, which neglect relations among spots. Third, existing algorithms achieve an excellent performance by sacrificing efficiency of methods, i.e. the time complexity is expensive. To address these mentioned limitations, we proposed a **j**oint learning nonnegative **m**atrix **f**actorization algorithm for **f**ast cell type **d**econvolution (aka jMF2D), which effectively integrates scRNA-seq and spatial transcriptomics data with network-based models. The overview of jMF2D is illustrated in Fig. 1, which consists of three major components, i.e. network construction, feature learning and cell type deconvolution. On network construction issue, jMF2D first learns similarity network of cell types with self-representation, and constructs spot spatial network with spatial location information, where indirected topological structure of cell types and spots are exploited. On the feature learning issue, jMF2D decomposes spatial expression profiles with NMF, where spot spatial network are integrated with the regularization strategy in machine learning, and similarity network of cell types are jointly learned. In this case, signatures and relations among them are incorporated into cell type deconvolution, bridging connection between scRNA-seq and spatial transcriptomics data. Finally, we formulate cell type deconvolution with an overall objective function, which is optimized with joint learning. Experiments demonstrate that jMF2D not only enhances performance of cell type deconvolution, but also dramatically saves the running time.

In all, major contribution of this study is summarized as

- First, jMF2D is a novel network-based joint learning algorithm for cell type deconvolution in spatial transcriptomics data, where topological structure of cell type and spatial networks provides additional information to dramatically enhance performance of algorithms. It also serves as a flexible framework for integrating multiomic data.
- Second, jMF2D is extra-ordinary fast by saving more than 90% running time of state-of-the-art algorithms, which is applicable for large-scale data. And, it also can serve as a pre-processing tool for spatial transcriptomics data.
- Third, extensive experiments demonstrate jMF2D is superior to baselines in terms of accuracy for cell type deconvolution with simulated and real spatial transcriptomics data. Furthermore, it also facilitates the identification of spatial domains and bio-marker genes.

## 2 Materials and methods

### 2.1 Mathematic model of jMF2D

Let $T\in R^{g\times n}$ denote expression profile of scRNA-seq for *g* genes and *n* cells with known annotation of cell types, and *X* be expression of spots in spatial transcriptomics data, where rows and columns correspond to genes and spots, respectively. K-nearest neighbor (KNN) is used to construct spot spatial network *W* with spatial coordinates of spots (the number of neighbors *k* is 6 for 10 × Genomics data, 8 for Slide-seq and Stereo-seq data, and 15 for imaging-based platforms), where weight $w_{ij}$ for the *i*-th and *j*-th spot is inverse proportional to the spatial Euclidean distance between them (denoted by $r_{ij}$), i.e. $w_{ij}={\left(r_{ij}\right)}^{-1}$. Degree of the *i*-th vertex of *W* is the sum of weights on edges connecting to it, i.e. $d_{i}=\sum_{j}w_{ij}$. Degree matrix of network *W* is the diagonal matrix with degree sequencing, i.e. $D=\mathrm{diag}(d_{1},\ldots ,d_{n})$, where *n* is the number of vertices. Laplacian matrix of network *W* is defined as $L_{W}=D-W$.

Given cell type signature *Y* of scRNA-seq data whose rows and columns denote genes and cell types respectively, we adopt self-representation learning to obtain cell type similarity network *Z* by minimizing the approximation, i.e.

(1) $$∥Y-YZ{∥}^{2},\;s.t.\;Z\geq 0,Z=Z^{{}^{\prime }},$$

where $Z^{{}^{\prime }}$ is the transpose of matrix *Z*, and ∥F∥ denotes $l_{2}$-norm of matrix *F*. We also expect matrix *Z* is sparse, and $L_{2,1}$-norm constraint is imposed. Therefore, Equation (1) is re-written as

(2) $$∥Y-YZ{∥}^{2}+∥Z{∥}_{2,1},\;s.t.\;Z\geq 0,Z=Z^{{}^{\prime }},$$

where $∥Z{∥}_{2,1}=\sum_{i}\sqrt{\sum_{j}z_{ij}^{2}}$.

jMF2D approximately decomposes expression profile of spatial transcriptomics *X* into product of two nonnegative low-rank matrix *B* and *F* with NMF (Lee and Seung 1999, Min *et al.* 2022) such that reconstruction error is minimized, i.e.

(3) $$∥X-BF{∥}^{2},\;s.t.\;B\geq 0,F\geq 0,$$

where *B* and *F* are the basis and coefficient matrix, respectively. Analogously, we also expect matrix *F* is sparse, and Equation (3) is re-written as

(4) $$∥X-BF{∥}^{2}+∥F{∥}_{2,1},\;s.t.\;B\geq 0,F\geq 0.$$

Notice that matrix *B* denotes association between genes and cell types, and we also expect to connect scRNA-seq with spatial transcriptomics to facilitate deconvolution of spots. Thus, we expect that, if a pair of cell types are similar in *Z*, they are also close in spatial transcriptomics, vice verse. It can be formulated as the trace optimization, i.e.

(5) $$\frac{1}{2}\sum_{ij}∥b_{.i}-b_{.j}{∥}^{2}z_{ij}=Tr(BL_{Z}B^{{}^{\prime }}),$$

where $b_{.i}$ is the *i*-th column of matrix *B*. Analogously, we also expect coefficient matrix *F* also reflects structure of spatial network *W*, i.e.

(6) $$\frac{1}{2}\sum_{ij}∥f_{.i}-f_{.j}{∥}^{2}w_{ij}=Tr(FL_{W}F^{{}^{\prime }}).$$

By combining Equations [(2), (4), (5), and (6)], the mathematic model of jMF2D is formulated as

(7) $$\begin{array}{l} L=∥X-BF{∥}^{2}+∥Y-YZ{∥}^{2}+\alpha (∥Z{∥}_{2,1}+∥F{∥}_{2,1})\\ +\beta Tr(BL_{Z}B^{{}^{\prime }})+\gamma Tr(FL_{W}F^{{}^{\prime }})\\ s.t.\;B\geq 0,F\geq 0,Z\geq 0,Z=Z^{{}^{\prime }}. \end{array}$$

The update rules for variables in Equation (7) are deduced (Supplementary Section: Optimization rules), available as supplementary data at *Bioinformatics* online.

### 2.2 Parameter selection

The mathematical model of jMF2D in Equation (7) involves three parameters, i.e. α, β, and γ, which are set as

- Parameter α controls importance of sparsity constraints of matrices, and we set α=1 in all experiments.
- Parameter β determines importance of the cell type similarity network, and we set $\beta \in [0,\frac{s}{k}]$, where *s* and *k* are the number of spots and cell types, respectively.
- Parameter γ determines importance of the spot spatial network, and we set γ≤1.

### 2.3 Simulated spatial transcriptomics dataset

Two strategies are used to generate simulated spatial dataset. Specifically, the first strategy generates dataset by merging several randomly selected cell types with predefined proportions for each spot without spatial information (Li *et al.* 2022), while the second strategy generates dataset by fixing the spot positions and randomly assigning single cells to each spot according to specified proportions, thereby incorporating spatial information (Ma and Zhou 2022).

### 2.4 Evaluation of performance for deconvolution

To fully evaluate performance of various algorithms for cell type deconvolution, four typical evaluation metrics are selected as criteria (Li *et al.* 2022), including PCC (Pearson Correlation Coefficient), SSIM (Structure Similarity Index Measure), JS (Jensen-Shannon divergence) and RMSE (Root Mean Square Error).

Specifically, PCC calculates correlation coefficient of between the predicted and ground truth cell types, and SSIM quantifies structure similarity between the predicted and ground truth cell types. RMSE measures deviation of cell types by computing Euclidean distance between expression profiles of the predicted and ground truth cell types. By treating expression profile of cell types as distribution, JS divergence calculates divergence between them as

(8) $$JS=\frac{1}{2}KL(P|\frac{1}{2}(P+Q))+\frac{1}{2}\left(Q|\frac{1}{2}(P+Q)\right)$$

where *P* and *Q* are the spatial probability distribution of the predicted and ground truth cell types, respectively.

## 3 Results

### 3.1 Benchmarking jMF2D against available baselines with simulated dataset

To comprehensively validate performance of jMF2D, the simulated spatial transcriptomics dataset (Li *et al.* 2022) is adopted to investigate whether it can precisely estimate abundance of cell types for each spot. Since the ground truth abundance of spots is known, four criteria are selected as measurements to quantify performance of various algorithms, including PCC, SSIM, JS, and RMSE. Eight popular algorithms, including Cell2location (Kleshchevnikov *et al.* 2022), RCTD (Cable *et al.* 2022), SpatialDWLS (Dong and Yuan 2021), SPOTlight (Elosua-Bayes *et al.* 2021), DestVI (Lopez *et al.* 2022), Redeconve (Zhou *et al.* 2023), and DSTG (Song and Su 2021), are deliberately selected as baselines because of their excellent performance. Notice that jMF2D makes use of spatial information for cell type deconvolution that is missed in simulated dataset. Here, we under-estimate performance of jMF2D by deliberately removing spatial information for a comparative comparison.

**Figure 2.**
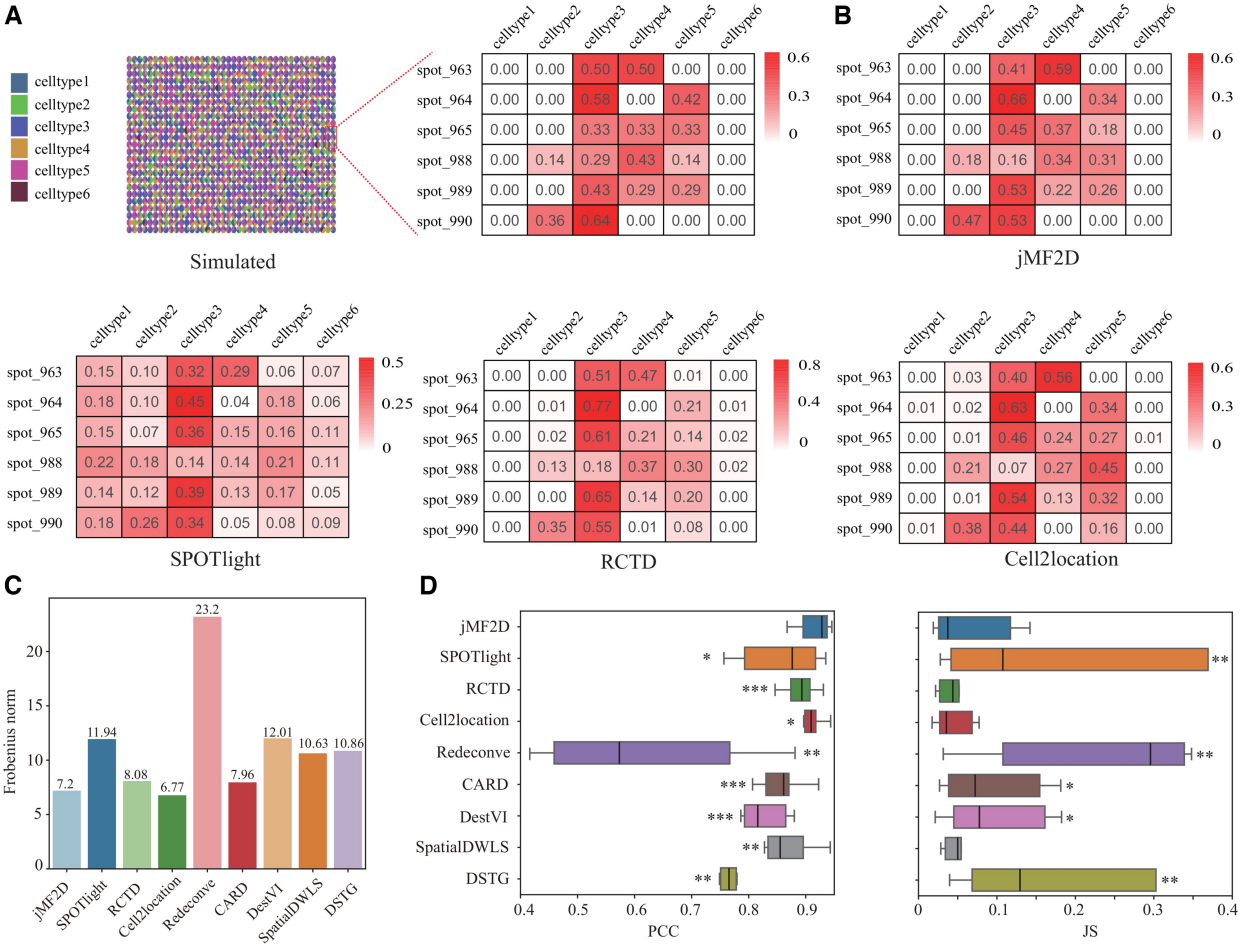
Performance of various algorithms for cell type deconvolution on the simulated dataset without spatial information. (A) Pie chart of the simulated spatial transcriptomics dataset, where different colors correspond to various cell types (left). And, zoomed region contains six spots with six cell types, where abundance of cell types is visualized as heatmap (right). (B) Visualization of abundance of cell types for the zoomed region estimated by the various algorithms. (C) Distance the predicted and ground truth abundance of cell types in the simulated dataset in terms of Frobenius norm. (D) Distribution of performance of various algorithms in terms of PCC (left) and JS (right), where significance is obtained with one-sided student’s t-test, and */**/*** denotes Benjamini-Hochberg (FDR-BH) adjusted *P*-value is <5.0E−1/5.0E−2/5.0E−3, respectively. Notably, the absence of a symbol indicates a lack of statistical significance.

**Figure 3.**
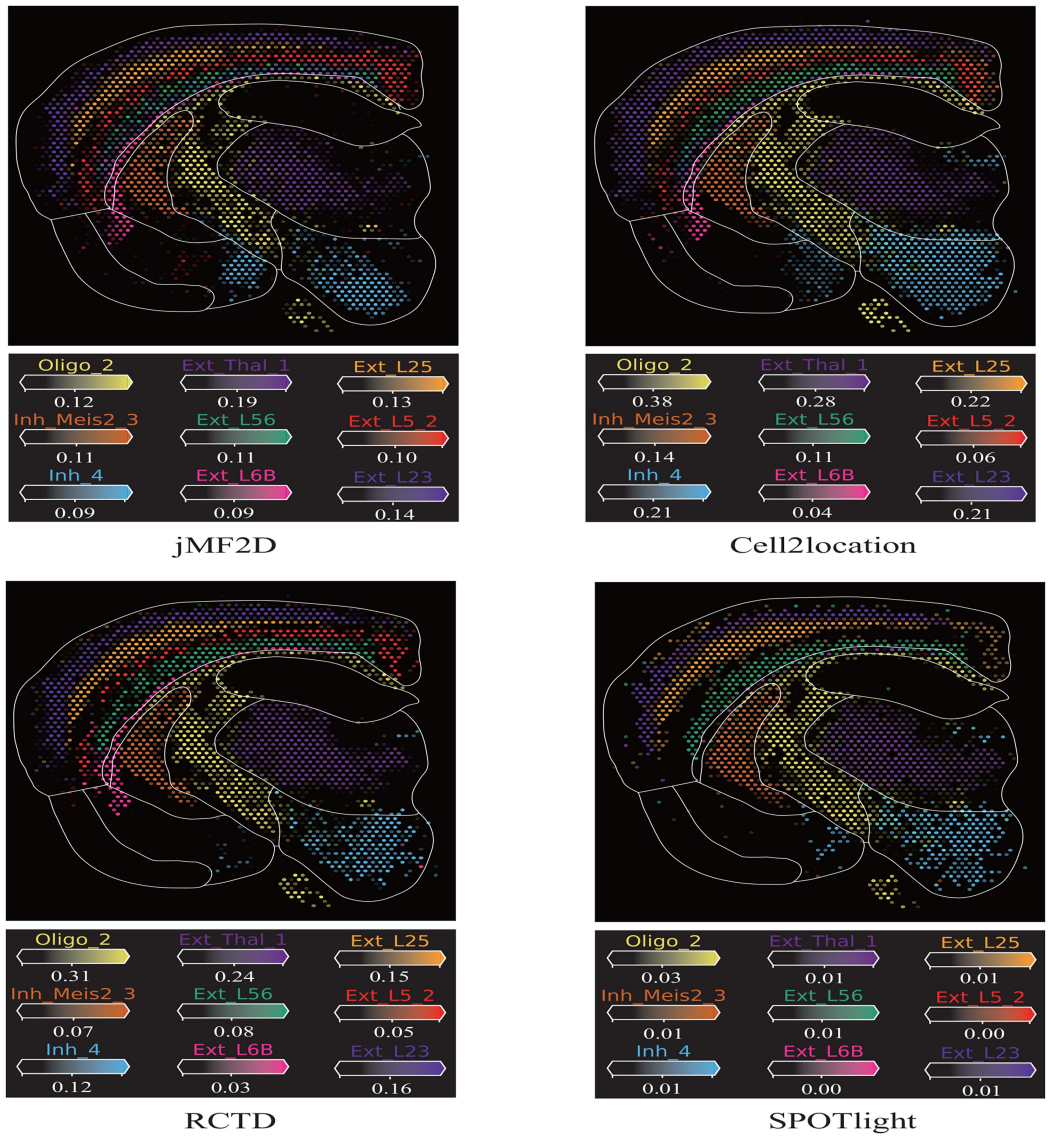
Visualization of results of region-specific cell types in the mouse brain obtained by various algorithms, where regions are enclosed by white lines.

**Figure 4.**
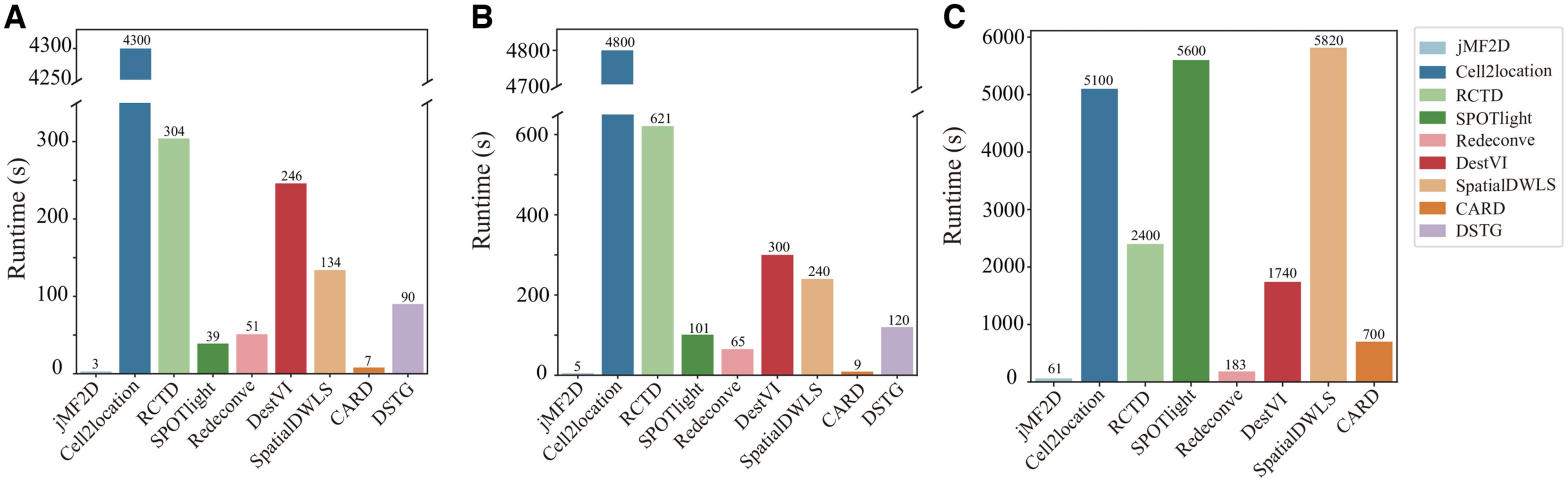
Running time of various algorithms on different datasets: (A) simulated dataset with 1000 spots and 19 850 genes (scRNA-seq data are with 3821 cells and 18 320 genes), (B) variant of simulated dataset with 1000 spots and 26 160 genes (scRNA-seq data are with 6948 cells and 19 909 genes), and (C) mouse brain dataset with 2987 spots (snRNA-seq data are with 13 936 cells and 21 799 genes), respectively.

**Figure 5.**
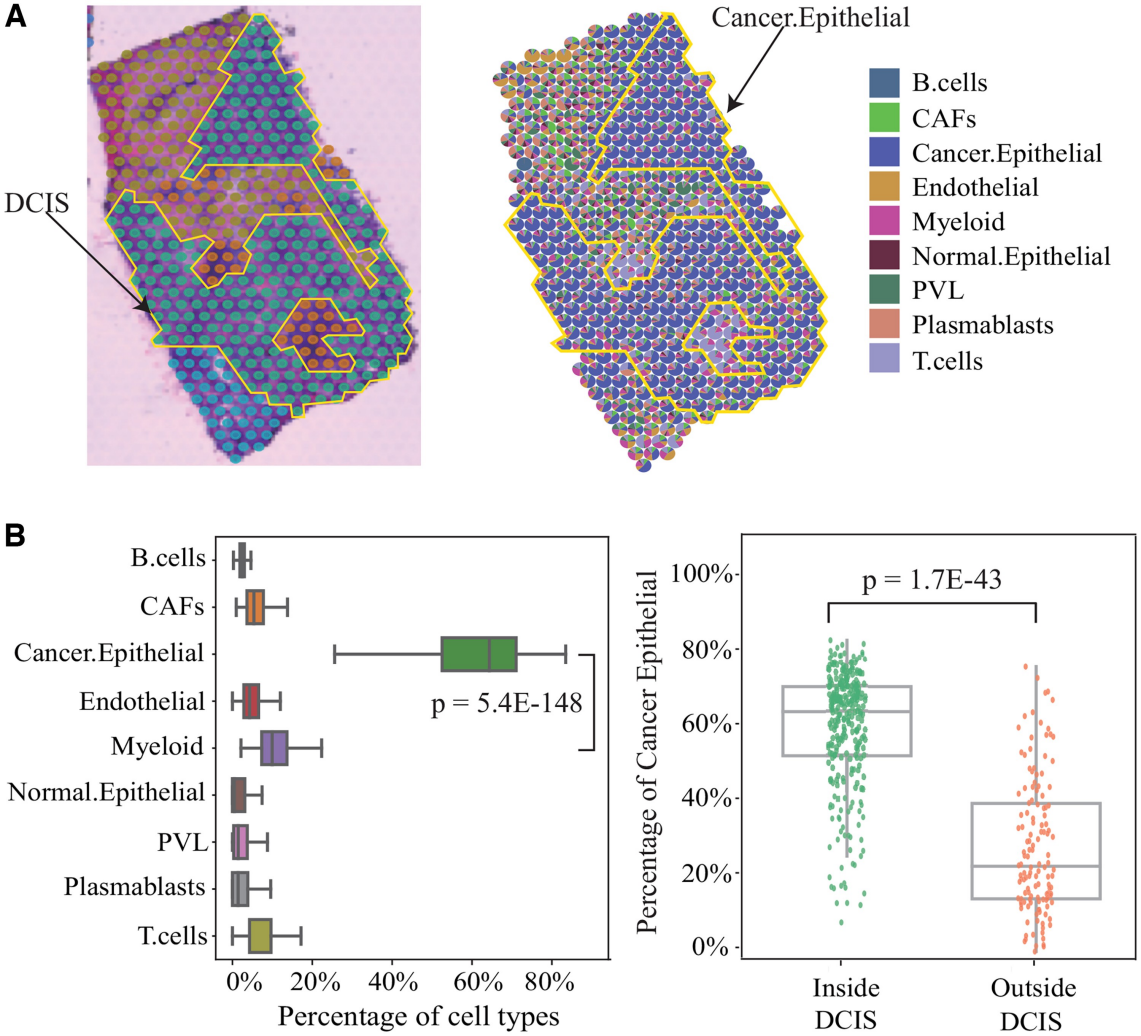
jMF2D precisely identifies cancer-related spatial domains and bio-marker genes from cancer spatial transcriptomics dataset: (A) H&E image of DCIS region of breast cancer(left), and spatial scatter pie chart (right), where the proportions of cell types for each spot are predicted by jMF2D, and (B) boxplot of proportions of cell types for each spot (left), and distribution of percentage of cancer epithelial cells in spots inside and outside of DCIS (significance is with the one-sided student’s *t*-test).

The simulated dataset is visualized in left panel of Fig. 2A, where zoomed region contains 6 spots (right). Performance of these algorithms for the zoomed region is shown in Fig. 2B (Fig. 1A, available as supplementary data at *Bioinformatics* online). It is easy to assert that SPOTlight is worse than others because values of the first column (cell-type-1) are much larger than 0, whereas those of others are close to 0. Then, we investigate performance of various algorithms for cell type deconvolution of the simulated dataset in terms of Frobenious norm as shown in Fig. 2C, where Cell2location, jMF2D, CARD, and RCTD are much better than that of others. PCC, JS, SSIM, and RMSE of various algorithms for simulated dataset are shown in Fig. 2D (Fig. 1B, available as supplementary data at *Bioinformatics* online), where jMF2D and Cell2location are similar, but much better than others. These results demonstrate that jMF2D accurately estimates mixture of cell types in simulated dataset since the difference between them is usually non-significant (one-sided student’s *t*-test). By comparing the cell type similarity network learned by jMF2D and the one constructed with Pearson correlation coefficient of profiles of cell types (Fig. 1C, available as supplementary data at *Bioinformatics* online), we find that jMF2D removes the influence of unrelated cell types. Performance of various algorithms on the variant of simulated dataset (generated with different values of parameter) further demonstrates the superiority of jMF2D for cell type deconvolution (Fig. 2, available as supplementary data at *Bioinformatics* online). Parameter analysis show that jMF2D is quite stable since ranges of parameters are wide (Fig. 3, available as supplementary data at *Bioinformatics* online). We also validate performance of various algorithms for deciphering abundance of cell types on the simulated dataset with spatial information, where jMF2D outperforms other baselines in terms of various metrics, indicating that incorporation of spatial information further enhances performance of deconvolution of cell types (Fig. 4, available as supplementary data at *Bioinformatics* online). Ablation study further proves that spatial and cell networks are important for cell type deconvolution (Fig. 5, available as supplementary data at *Bioinformatics* online).

What we want to point out is that, even though jMF2D is under-estimated, it still achieves competitive performance with the best baseline. There are several good reasons to explain why the proposed algorithm is more accurate for cell type deconvolution. First, jMF2D removes independence assumption of cell types in the current algorithms, where relations among cell types facilitate the estimation of abundance of cell types. Second, jMF2D jointly learns features of cell types and spots, as well as topological structure of cell type networks, where structure of cell type network can serve as prior information for features of cell types and spots. Finally, spatial information is also incorporated, which is also critical for cell type deconvolution.

### 3.2 Benchmarking jMF2D against baselines with mouse brain dataset

The mouse brain dataset, consisting of diverse neural cell types organized in a well-characterized spatial architecture, is selected for experiments. The manually annotations of 59 cell types are from Kleshchevnikov *et al.* (2022), we selected one slice of mouse brain dataset and two snRNA-seq datasets to decipher the nine region-specific cell types (Fig. 3).

**Figure 6.**
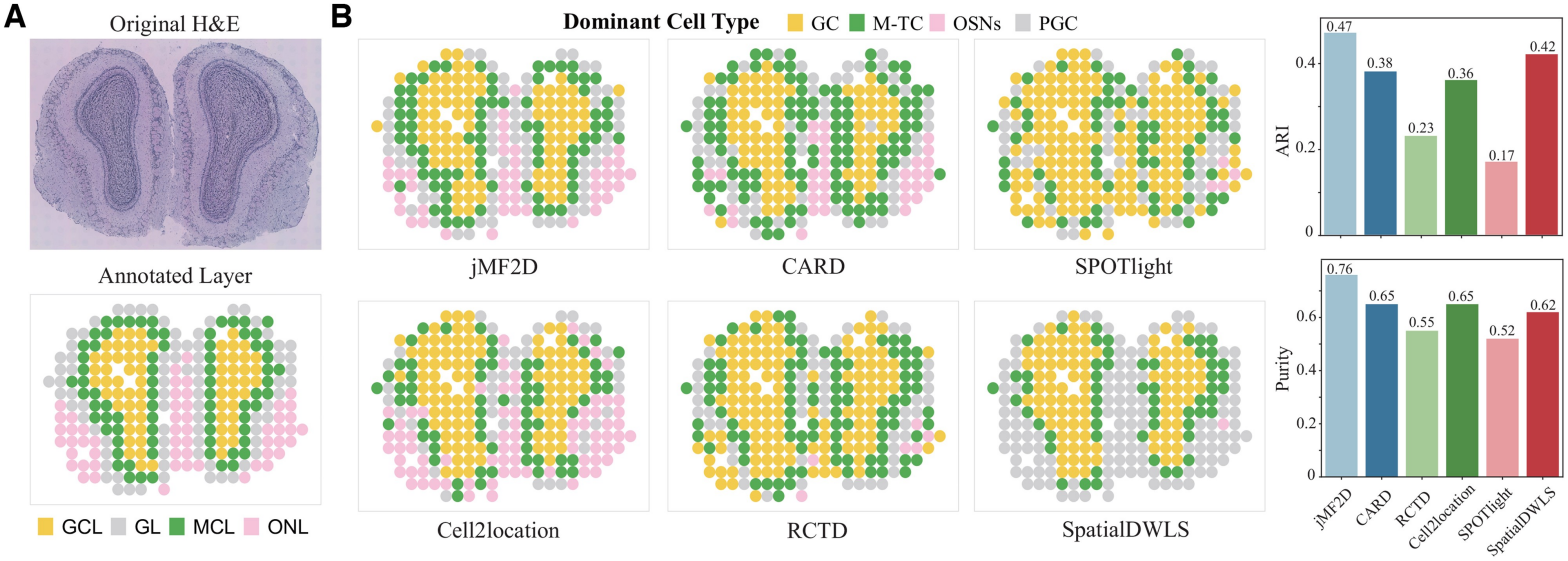
Results of jMF2D on the mouse olfactory bulb (MOB) dataset generated by Legacy platform: (A) H&E staining of the olfactory bulb dataset and the four anatomical layers from inside to outside, including GCL (Granule Cell Layer), MCL (Mitral Cell Layer), GL (Glomerular Layer), and ONL (Nerve Layer), and (B) performance of different algorithms on the identification of spatial domains (left), and ARI and Purity of spatial domains identified by various approaches (right), respectively.

We visualize the typical nine cell types identified by various algorithms with cell type deconvolution as shown in Fig. 3 (Fig. 6, available as supplementary data at *Bioinformatics* online). From these panels, we find that only jMF2D and Cell2location precisely identify these nine cell types, whereas others fail to precisely identify some of them, demonstrating that these two algorithms accurately decipher mixture of cell types of spots of mouse brain dataset. For instance, SPOTlight fails to correctly identify the Ext_L5_2 and Ext_L6B, whereas Cell2location and jMF2D successfully recognize these cell types. In details, jMF2D and Cell2location identify Ext_L5_2 with region coherence, whereas RCTD fails to identify it. Additionally, we compare the spatial distributions of cell types and their corresponding bio-marker genes to quantify performance of various algorithms, where jMF2D is better than baselines. (Fig. 7, available as supplementary data at *Bioinformatics* online). These results demonstrate that jMF2D also precisely estimates abundance of cell types of spots of biological spatial transcriptomics datasets, facilitating the identification of cell types.

### 3.3 jMF2D significantly accelerates speed of cell type deconvolution

Advances in spatial transcriptomics technology generate large-scale datasets, posing a great challenge on efficiency of algorithms for cell type deconvolution. And, Fig. 4 plots the running time of various algorithms on various datasets, where panel A is for the simulated dataset, B for variant version of simulated dataset, and C for mouse brain dataset, respectively. From these panels, it is easy to conclude that jMF2D is much faster than state-of-the-art baselines, followed by CARD, for all these three datasets. Specifically, the running time of jMF2D is 3.0 s, whereas that of Cell2location is 4300.0 s for the simulated dataset. The reason why Cell2location is time-consuming is that the probabilistic model requires expensive complexity for training. These results demonstrate that jMF2D is efficient regardless of datasets. Furthermore, we also validate scalability of jMF2D with two additional large-scale datasets, where the simulated dataset is extended by increasing the number of spots from 1000 to 11 000, and the mouse cerebellum dataset by varying the number of spots from 1000 to 11 588. The running time of jMF2D for simulated dataset is shown in Fig. 8A, available as supplementary data at *Bioinformatics* online, where the running time increases as the number of spots increases. Furthermore, the running time of jMF2D linearly increases in terms of the number of spots. Specifically, the running time of jMF2D is 6.33 s for 1000 spots, and it increases to 191.77 s if the number of spots is 11 000. It demonstrates that jMF2D is efficient for addressing large-scale simulated dataset. Then, we apply jMF2D to the large-scale mouse cerebellum dataset (Fig. 8B, available as supplementary data at *Bioinformatics* online), where performance of jMF2D is consistent with simulated dataset. In details, jMF2D only takes 1.56 s for mouse cerebellum dataset with 1000 spots, and requires 15.77 s if the number of spots is 11 588. These results prove that jMF2D is efficient and applicable for large-scale datasets.

### 3.4 jMF2D facilitates the identification of cancer-related spatial domains and bio-marker genes from cancer spatial transcriptomics dataset

Low-resolution spatial transcriptomics techniques, such as 10× Visium, provide whole-genome-wide gene expression data at spot level that consists of mixture of multiple cell types, hindering the down-stream analysis. Therefore, we further validate performance of jMF2D by investigating whether cell type deconvolution enhance the identification of cancer-related spatial domains and bio-marker genes of cancers (Min *et al.* 2025).

Figure 5A (left) visualizes H&E image of breast cancer (Wu *et al.* 2021), where the manually annotated DCIS (Ductal Carcinoma In Situ) region is labeled with the yellow and solid lines. jMF2D deciphers cell types for each spot is shown in the right panel of Fig. 5A, where pie charts of spots illustrates the proportions of cell types of spots, revealing the high spatial heterogeneity of breast cancer. Interestingly, we find that cancer epithelial cells are more significantly enriched than others in DCIS, which is consistent with assertion in Wu *et al.* (2021). In details, the left panel of Fig. 5B shows distributions of percentages of cell types of spots within DCIS regions, demonstrating that cancer epithelial cells dominate DCIS. Then, to check whether cancer epithelial cells are spatial region-specific, we compare the distribution of cancer epithelial inside and outside of DCIS (Fig. 5B right), where cancer epithelial cells are more abundant within DCIS (*P* = 1.7E−43, one-sided student’s *t*-test). These results demonstrate that deconvolution of cell types by jMF2D provides clues for biologists for down-stream analysis.

Then, we validate whether deconvolution of cell types facilitate the identification of spatial domains, where the Leiden algorithm is adopted for clustering of dataset deciphered by various algorithms (Fig. 9A, available as supplementary data at *Bioinformatics* online). Interestingly, performance of algorithms for spatial domains on the dataset deciphered by jMF2D is much better than those by others, demonstrating that jMF2D is more accurate to estimate abundance of cell types of spots. In details, ARI and purity of algorithms on the dataset processed by jMF2D are higher than others. Furthermore, spatial variable genes (SVGs) within spatial domains exhibits a coherent expression pattern with respect to the DCIS region, demonstrating that these genes serve as bio-marker genes (Fig. 9B, available as supplementary data at *Bioinformatics* online). The Moran’s *I* and Geary’s *C* statistics further prove that these SVGs are highly related to breast cancer. Then, we compare the cell type similarity networks learned by jMF2D and constructed by Pearson correlation coefficients of cell types (Fig. 9C, available as supplementary data at *Bioinformatics* online), where relations of cell types are precisely identified by jMF2D, demonstrating that cell type deconvolution also facilitates the exploitation of relations among cell types. Additionally, analysis of the rest regions of breast cancer dataset is also executed (Figs 10–14, available as supplementary data at *Bioinformatics* online), showing that jMF2D is also superior for cell type deconvolution.

### 3.5 jMF2D is applicable for spatial transcriptomics datasets generated by various platforms

Previous experiments only validate performance of jMF2D for 10× Genomics Visium platform, and then we check whether it is sensitive to specific platforms by selecting mouse olfactory bulb (MOB) spatial transcriptomics dataset generated from Legacy ST platform. The H&E image of MOB is shown in Fig. 6A, which comprises four primary anatomical layers, i.e. GCL, MCL, GL, and ONL.

Following Ma and Zhou (2022), we only retain the most predominant cell type for each spot as shown in Fig. 6B, where jMF2D accurately identifies the layered structure. Specifically, jMF2D precisely identifies the boundary distribution of M-TC type, which is consistent with MCL. Furthermore, ARI and purity results demonstrate that jMF2D outperforms these baselines in identifying these four layers (right panel of Fig. 6B). Even though these baselines also reveal certain patterns of heterogeneity (Fig. 15A, available as supplementary data at *Bioinformatics* online), jMF2D accurately differentiates MCL and GL layers, with mitral/tufted cells and periglomerular cells exhibiting distinct distributions across these layers. However, baselines cannot precisely address this issue. Then, we examine consistency between spatial distribution of cell types identified by jMF2D and their corresponding bio-marker genes (Fig. 15B, available as supplementary data at *Bioinformatics* online). Interestingly, we find that distribution of cell types is highly consistent with the spatial distribution of their bio-marker genes, showing that jMF2D leverages topological structure of spatial and cell similarity network to model dataset generated by various platforms.

Finally, we also explore cell type similarity network learned by jMF2D for various platforms (Fig. 15C, available as supplementary data at *Bioinformatics* online), which is consistent with that of 10× Genomics Visium platform, showing jMF2D is insensitive to platforms. Analogously, we also exploit cell similarity network and investigate importance of networks with an ablation study for jMF2D (Fig. 16, available as supplementary data at *Bioinformatics* online). These results demonstrate that spatial and transcriptomic networks are critical for cell type deconvolution, which is also the reason why jMF2D is superior to current baselines. Moreover, network-based model is also promising for cell type deconvolution, which is applicable to datasets generated from various platforms.

## 4 Conclusion

Spatial transcriptomics technologies enable acquiring spatial locations and gene expression profiles of spots, providing a great opportunity to exploit structure and mechanisms of biological systems. However, the limitation of resolutions of spots hampers the down-stream analysis since the expression of genes is measured at the spot level, rather than cell level. Deconvolution of cell types of spots integrates spatial transcriptomics and scRNA-seq data to elucidate spatial distribution of cell types, facilitating the understanding of structure and functions of biological systems.

In this study, we enhance and accelerate cell type deconvolution by developing a fast and accurate network-based algorithm jMF2D by exploiting topological structure of cell type and spatial networks. jMF2D leverages the topological information of networks to facilitate the estimation of cell type abundance of spots. Experiments demonstrate that jMF2D not only enhances and accelerates cell type deconvolution of datasets generated by various platforms, but also facilitates the identification of bio-markers, such as spatial domains and bio-marker genes. We see ample opportunities to further improve the proposed algorithm. First, jMF2D only integrates spatial transcriptomics and scRNA-seq data, and additional information can be integrated to further exploit cell type relations. Second, morphological images is also promising for spatial transcriptomics data (Min *et al.* 2024).

## Supplementary Material

btaf419_Supplementary_Data

## Data Availability

All datasets are downloaded from the public accessions. The simulated dataset is available at here. The simulated dataset with spatial information can be accessed here. The mouse cerebellum dataset is available at here. The mouse brain snRNA-seq and spatial transcriptomics datasets are available at E-MTAB-11115 and E-MTAB-11114. The human breast cancer datasets are available at Broad Institute and Zenodo, respectively. The mouse olfactory bulb data can be accessed from the following source: MOB. jMF2D is coded by python, and it is freely available at here. An archived version of the code is available on Zenodo (DOI: 10.5281/zenodo.15813049).
