# Supplementary material for: Enhancing and accelerating cell type deconvolution of large-scale spatial transcriptomics slices with dual network model
Source: Bioinformatics. 2025 Jul 24;41(8):btaf419. doi: 10.1093/bioinformatics/btaf419 (PMC12341680; doi:10.1093/bioinformatics/btaf419)
Supplement: btaf419_Supplementary_Data [file btaf419_supplementary_data.pdf]

Mathematic model of jMF2D

jMF2D performs cell type deconvolution by exploiting topological structure of dual networks of cell types and spatial spots, which involves two critical issues, i.e., construction of networks, and feature learning.

Objective function

On the construction of cell type similarity network, let matrix T be the profile of scRNA-seq data matrix with g genes and c cells. First, we estimate the cell-type-specific expression profile $Y_{i,k}$ as the average expression of gene i across all cells j within cell type k as follows:

$$Y_{i,k} = \frac{1}{j_k} \sum_{j=1}^{j_k} T_{i,j} \tag{1}$$

where j_k is the total number of cells in the k-th cell type. Then, the self-representation learning (Hu et al., 2017) is adopted to obtain cell-type similarity Z, which is formulated as

$$\|Y - YZ\|^2 \quad s.t. Z \geq 0, Z = Z' \tag{2}$$

here the first constrain requires non-negativity of cell type similarity, and the last one ensures symmetry of similarity. Furthermore, we also expect the cell type similarity Z is sparse so the $L_{2,1}$-norm constraint is imposed on it, i.e.,

$$\|Y - YZ\|^2 + \|Z\|_{2,1} \quad s.t. Z \geq 0, Z = Z'. \tag{3}$$

On the feature learning of cell type similarity network, jMF2D obtains the composition of cell types for each spot by decomposing on the spatial transcriptomics data matrix X with nonnegative matrix factorization (NMF) (Lee and Seung, 1999). Analogously, we also expect F to be sparse, which is formulated as

$$\|X - BF\|^2 + \|F\|_{2,1} \quad s.t. B \geq 0, F \geq 0. \tag{4}$$

The local preservation theory demonstrates that if a pair of cell types are similar in Z, they are also close in spatial transcriptomics, vice verse(Xu et al., 2010). Luckily, it is re-formulated as

$$\begin{aligned} &\frac{1}{2} \sum_{i,j=1} \|\boldsymbol{b}_{.i} - \boldsymbol{b}_{.j}\|^2 z_{ij} \\ &= \sum_{i=1} \boldsymbol{b}'_{.i} \boldsymbol{b}_{.i} d_{ii} - \sum_{i,j=1} \boldsymbol{b}'_{.i} \boldsymbol{b}_{.j} z_{ij} \\ &= Tr(BDB') - Tr(BZB') \\ &= Tr(BL_Z B') \end{aligned} \tag{5}$$

where $\boldsymbol{b}_{.i}$ is the i-th column of matrix B, D is a diagonal matrix with degree sequence of Z, and L_Z is Laplacian matrix of Z, $L_Z = D - Z$.

On the construction of spot spatial network, jMF2D employs K-nearest neighborhood (KNN) algorithm (the number of nearest neighbors set as 6), where the edge weights in spatial network W is proportional to Euclidean distance of spatial information of spots, i.e., $1/r_{ij}$. Analogously, we also expect features of spots F also reflects the topological structure of spot spatial network, which is also formulated as trace optimization, i.e.,

$$\frac{1}{2} \sum_{i,j=1} \|\boldsymbol{f}_{.i} - \boldsymbol{f}_{.j}\|^2 w_{ij} = Tr(FL_W F'). \tag{6}$$

By joining equation (3), (4), (5) and (6), the final objective function of jMF2D is re-written as

$$\begin{aligned} \mathcal{L} &= \|X - BF\|^2 + \|Y - YZ\|^2 + \alpha(\|Z\|_{2,1} + \|F\|_{2,1}) \\ &+ \beta Tr(BL_Z B') + \gamma Tr(FL_W F') \\ &s.t. \quad B \geq 0, F \geq 0, Z \geq 0, Z = Z' \end{aligned} \tag{7}$$

where parameter α determines the weight of the $L_{2,1}$-norm, β determines the weight of preserving the cell similarity Z topology in the spatial transcriptomics, and γ determines the weight of preserving the spatial topology information.

Optimization rules

Due to the non-convex nature of the objective function in Eq.(7), we optimize it with alternative strategy, i.e., we update one variable by fixing others, and this procedure continues until it converges or the maximal number of iterations reaches.

Update variable Z

By removing irrelevant terms of Z from the objective function, Eq.(7) is equivalent to the following problem, i.e.,

$$\min_Z \|Y - YZ\|^2 + \alpha\|Z\|_{2,1}. \tag{8}$$

By setting alternative variable C for Z, Eq.(8) is further transformed into the following problem, i.e.,

$$\begin{aligned} &\min_{Z,C} \|Y - YZ\|^2 + \alpha\|C\|_{2,1} + \langle T_1, Z - C\rangle + \delta_1\|Z - C\|^2 \\ &\qquad s.t. \quad Z = C \end{aligned} \tag{9}$$

where $\langle\cdot\rangle$ represent the dot product, α is a weighting parameter, T_1 is the Lagrange multiplier, and $\delta_1 > 0$ is the penalty coefficient. Notice that variables Z and C can be optimized with ADMM (Boyd et al., 2011) . Specifically, the augmented Lagrangian function of Eq.(9) is deduced as

$$\min_{Z,C} \|Y - YZ\|^2 + \alpha\|C\|_{2,1} + \delta_1\|Z - C + \frac{T_1}{\delta_1}\|^2, \tag{10}$$

which can be reformulated into the following sub-problems

$$\min_Z \|Y - YZ\|^2 + \delta_1\|Z - C + \frac{T_1}{\delta_1}\|^2, \tag{11}$$

$$\min_C \quad \alpha\|C\|_{2,1} + \delta_1\|Z - C + \frac{T_1}{\delta_1}\|^2, \tag{12}$$

$$T_1 = T_1 + \delta_1(Z - C). \tag{13}$$

Eq. (11) is convex by regarding Z. By setting the partial derivative with respect to Z to zero, the update rule for Z is formulated as

$$Z = Z \odot \frac{Y'Y + \delta_1 C - T_1}{Y'YZ + \delta_1 Z}, \tag{14}$$

where $\odot$ is the Hadamard product. Analogously, the update rule for C is deduced as (Nie et al., 2010)

$$C = C \odot \frac{\delta_1 Z + T_1}{\alpha D_1 C + \delta_1 C}, \tag{15}$$

where D_1 is a diagonal matrix with the i-th diagonal element as $d_{ii} = \frac{1}{2\|\boldsymbol{c}_{i.}\|_2}$ ($\boldsymbol{c}_{i.}$ is the row vector of C).

Update variable B

For variable B, the nonnegative constraint is addressed by Larange method. Specifically, let ϕ_{ij} be the Larange multipler for the constraint b_{ij}. Eq.(7) is derived as

$$\min_{B} \|X - BF\|^2 + \beta Tr(BL_Z B^T) + Tr(\Phi B). \tag{16}$$

According to the Karush-Kuhn-Tucker conditions, by setting the derivative of B and $\phi_{ij} b_{ij} = 0$, the update rule for variable B is obtained as

$$B = B \odot \frac{XF^{'}}{BFF' + \frac{\beta}{2}(BL_Z + BL'_Z)}. \tag{17}$$

Update variable F

Analogous to variable B, the nonnegativity constraint on variable F is also addressed by Larange method. Let Ψ be the Larange multipliers for variable F, Eq.(7) is equivalent to the following function

$$\min_{F} \|X - BF\|^2 + \alpha\|F\|_{2,1} + \gamma Tr(FL_W F') + Tr(\Psi F). \tag{18}$$

Similar to the solution process for variable Z, we can further obtain the augmented Lagrangian function

$$\begin{aligned} \min_{F,E} \|X - BF\|^2 + \alpha\|E\|_{2,1} + \gamma Tr(EL_W E') \\ +\delta_2\|F - E + \frac{T_2}{\delta_2}\|^2 + Tr(\Psi F), \end{aligned} \tag{19}$$

which can be decomposed into following sub-problems

$$\min_{F} \|X - BF\|^2 + \delta_2\|F - E + \frac{T_2}{\delta_2}\|^2 + Tr(\Psi F), \tag{20}$$

$$\min_{E} \quad \alpha\|E\|_{2,1} + \gamma Tr(EL_W E') + \delta_2\|F - E + \frac{T_2}{\delta_2}\|^2, \tag{21}$$

$$T_2 = T_2 + \delta_2(F - E), \tag{22}$$

where α and γ are weighting parameters, T_2 is the Lagrange multiplier, and $\delta_2 > 0$ is the penalty coefficient. By setting the derivative of F and $\Psi_{ij} f_{ij} = 0$, the update rule for F is formulated as

$$F = F \odot \frac{B^{'}X + \delta_2 E}{B'BF + \delta_2 F + T_2}, \tag{23}$$

and

$$E = E \odot \frac{\delta_2 F + T_2}{\alpha D_2 E + \delta_2 E + \frac{\gamma}{2}(EL_W + EL'_W)}. \tag{24}$$

where D_2 is a diagonal matrix with the i-th diagonal element as $d_{ii} = \frac{1}{2\|\boldsymbol{e}_{i.}\|_2}$ ($\boldsymbol{e}_{i.}$ is the row vector of E).

Clustering evaluation metrics

In spatial domain identification, we compare the results of different algorithms in identifying domains based on H&E staining using (Adjusted Rand Index) ARI and Purity (Ma and Zhou, 2022). Specifically, ARI is defined as:

$$ARI(\Omega, C) = \frac{\sum_{ls}\binom{n_{ls}}{2} - [\sum_l \binom{a_l}{2}\sum_s \binom{b_s}{2}]/\binom{n}{2}}{\frac{1}{2}[\sum_l \binom{a_l}{2} + \sum_s \binom{b_s}{2}] - [\sum_l \binom{a_l}{2}\sum_s \binom{b_s}{2}]/\binom{n}{2}} \tag{25}$$

where $\Omega = (w_1, w_2, \cdots, w_n)$ and $C = (c_1, c_2, \cdots, c_n)$ donote the identified spots and true labeled spots, respectively. n represents the total number of spots. And l and s enumerate the domains, with $l = 1,\cdots,r$ and $s = 1,\cdots,k$ where r and k are the number of identified domains and true labeled domains, respectively. n_{ls} is the number of times where the i-th spot belongs to the domain l in the identified domain labeling and j-th spot belongs to the domain s in the true domain labeling. $a_l = \sum_s n_{ls}$, $b_s = \sum_l n_{ls}$ and $\binom{*}{*}$ represents the binomial coefficient. Subsequently, Purity is defined as:

$$Purity(\Omega, C) = \frac{1}{n}\sum_{w\in\Omega} max|w \cap c| \tag{26}$$

Experimental settings

In this section, we provide a detailed description of the data preprocessing procedures and parameter settings for the algorithm across different experiments.

Data preprocessing

All our experiments follow a unified data preprocessing procedure. First, we extract the intersecting genes between the single-cell and spatial transcriptomics data. Next, the following processing steps are applied to each dataset separately:

1) For the single-cell data, the top 5000 highly variable genes (HVGs) are identified with Seurat (version 3.0), followed by standard preprocessing steps in Scanpy, including normalize_total, log1p, and scale. Finally, we obtain the expression profiles of cell types corresponds to the average profiles of cells within them.

2) For the spatial transcriptomics data, we apply the same standard Scanpy preprocessing pipeline to obtain the expression profiles of spots.

Parameter settings

In this section, we list the specific parameter settings for each experiment to facilitate reproducibility.

Table 1. Parameter settings for different experiments

datasets	α	β	γ	max_iter
simulated dataset 1	1	1	1	1000
simulated dataset 2	1	1	1	500
simulated dataset with spatial information	1	1	1	1000
mouse brain	1	1	0.5	500
mouse cerebellum	1	1	1	500
human breast cancer	1	1	1	1000
mouse olfactory bulb	1	1	1	1000

Datasets employed for different experiments

In this section, we list the links to single-cell and spatial transcriptomics datasets used in different experiments.

Table 2. Datasets employed for different experiments

**Experiments**	**Datasets**	**Link**
Benchmarking on simulated datasets	simulated dataset	here
Benchmarking on simulated datasets with spatial information	simulated dataset with spatial information	here
Benchmarking on mouse brain datasets	snRNA-seq	here
	ST data	here
Runtime evaluation of large-scale ST data	mouse cerebellum	here
Identification of cancer-related spatial domains in human breast cancer	scRNA-seq	here
	ST data	here
Applications on various spatial transcriptomics platforms	mouse olfactory bulb	here

References

Boyd, S., Parikh, N., Chu, E., Peleato, B., Eckstein, J., et al. (2011). Distributed optimization and statistical learning via the alternating direction method of multipliers. *Foundations and Trends® in Machine learning*, 3(1):1–122.

Hu, R., Zhu, X., Cheng, D., He, W., Yan, Y., Song, J., and Zhang, S. (2017). Graph self-representation method for unsupervised feature selection. *Neurocomputing*, 220:130–137.

Lee, D. D. and Seung, H. S. (1999). Learning the parts of objects by non-negative matrix factorization. *nature*, 401(6755):788–791.

Ma, Y. and Zhou, X. (2022). Spatially informed cell-type deconvolution for spatial transcriptomics. *Nature biotechnology*, 40(9):1349–1359.

Nie, F., Huang, H., Cai, X., and Ding, C. (2010). Efficient and robust feature selection via joint $\ell 2,1$-norms minimization. *Advances in neural information processing systems*, 23.

Xu, Y., Song, F., Feng, G., and Zhao, Y. (2010). A novel local preserving projection scheme for use with face recognition. *Expert Systems with applications*, 37(9):6718–6721.

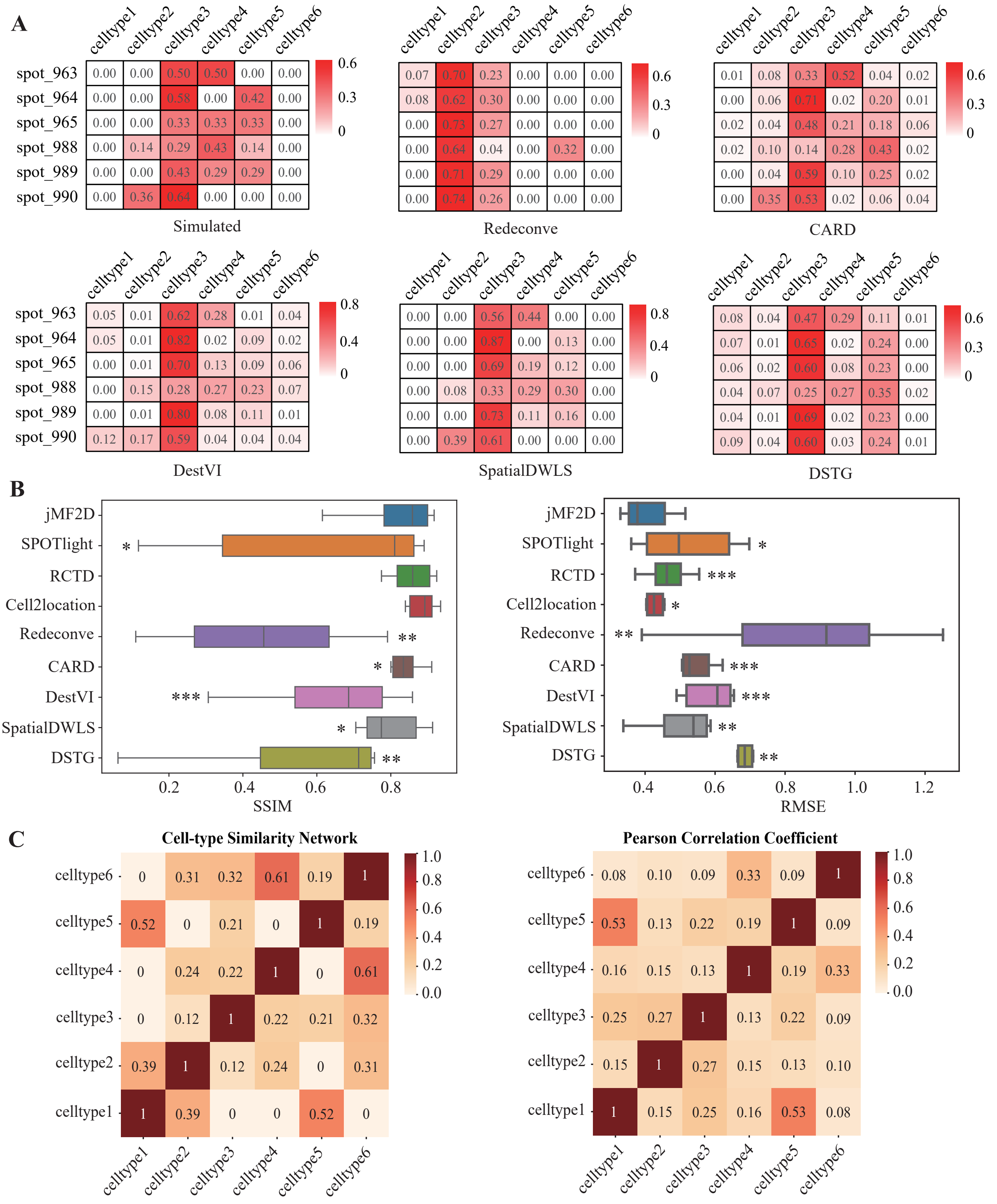

Fig. S1. Performance of various algorithms on the simulated dataset. (**A**) The heatmap of six selected spots for the various algorithms (**B**) The distribution of SSIM and RMSE of various algorithms on the simulated dataset, where are calculated between the predicted cell type proportions and the ground truth. Pairwise significance tests were conducted between algorithms, where significance is obtained with one-sided student's t-test, and */**/*** denotes Benjamini-Hochberg (FDR-BH) adjusted p-value is less than 5.0E-1/5.0E-2/5.0E-3, respectively. Notably, the absence of a symbol indicates a lack of statistical significance. (**C**) Heatmap of cell type similarity network and Pearson Correlation Coefficient between cell types on the simulated dataset.

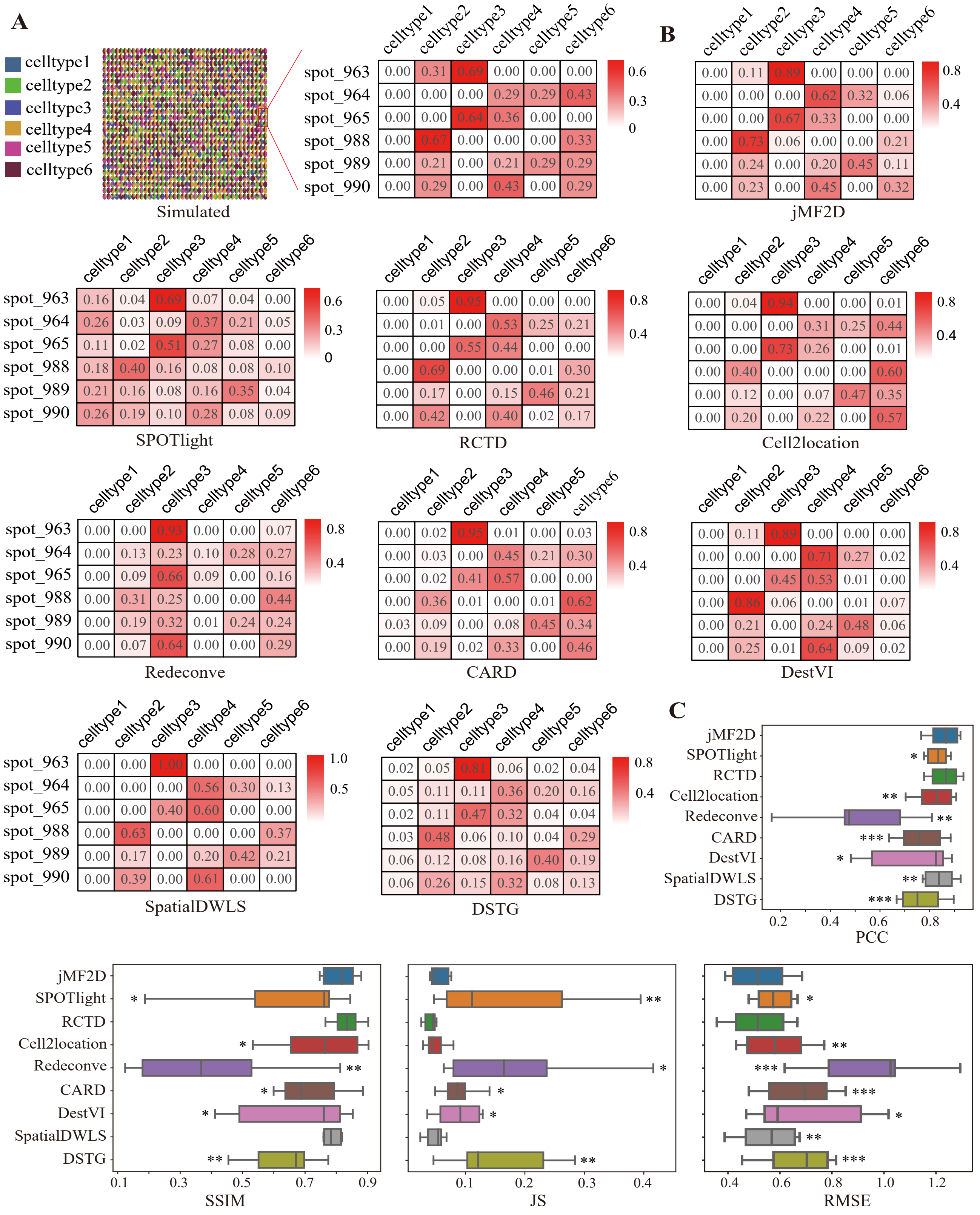

Fig. S2. Performance of various algorithms on variants of the simulated dataset generated from different scRNA-seq data of the same tissue. (**A**) The spatial pie chart and the heatmap of the selected 6 spots. (**B**) Visualization of cell abundance in the zoomed-in region estimated by different algorithms. (**C**) Performance of algorithms for cell type distribution estimated by different algorithms against ground truth using the metrics PCC, SSIM, JS, and RMSE, respectively. Pairwise significance tests were conducted between algorithms, where significance is obtained with one-sided student's t-test, and */**/*** denotes Benjamini-Hochberg (FDR-BH) adjusted p-value is less than 5.0E-1/5.0E-2/5.0E-3, respectively. Notably, the absence of a symbol indicates a lack of statistical significance.

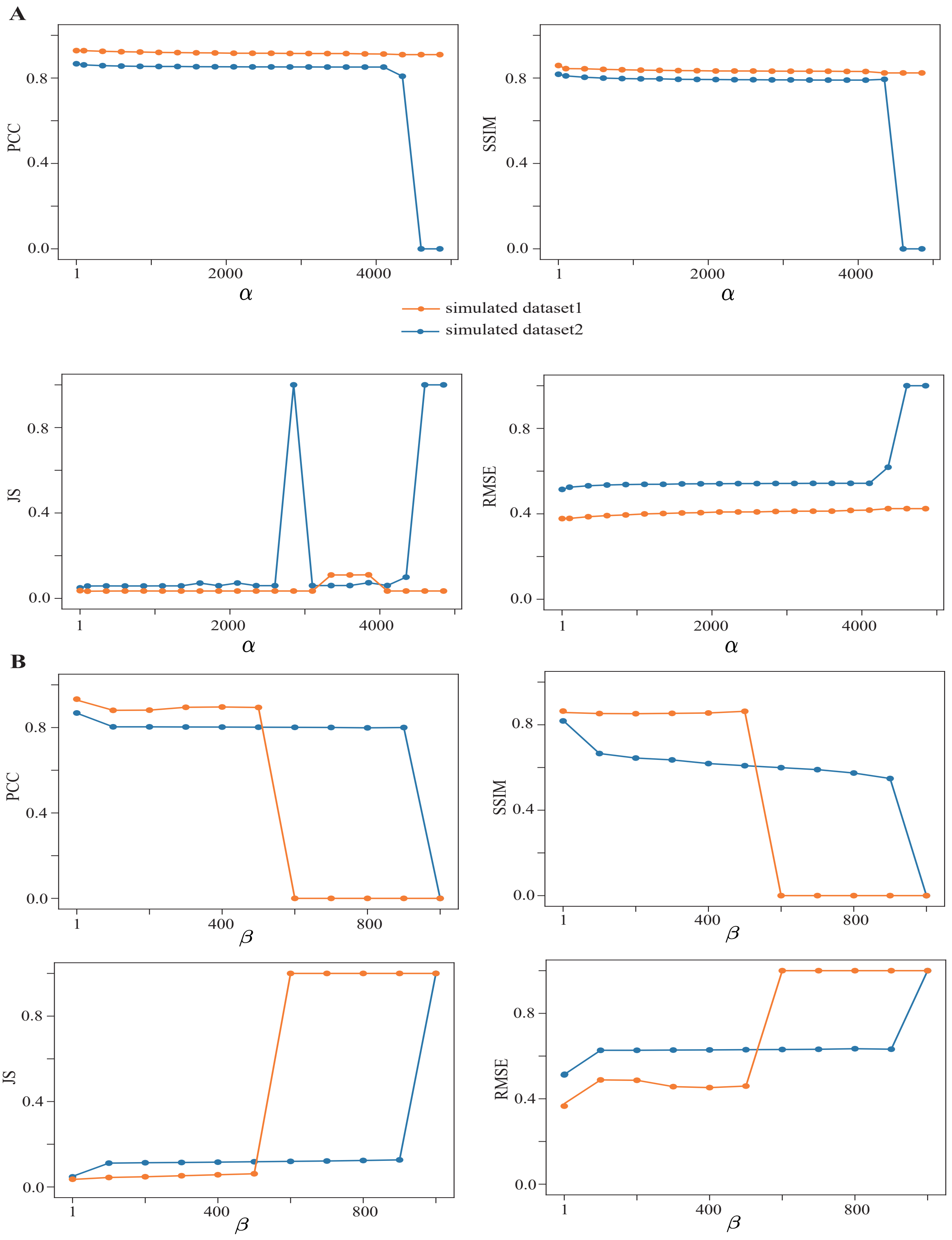

Fig. S3. Parameter analysis of jMF2D. (**A**) performance vs parameter α for different simulated data. (**B**) performance vs parameter β for different simulated data.

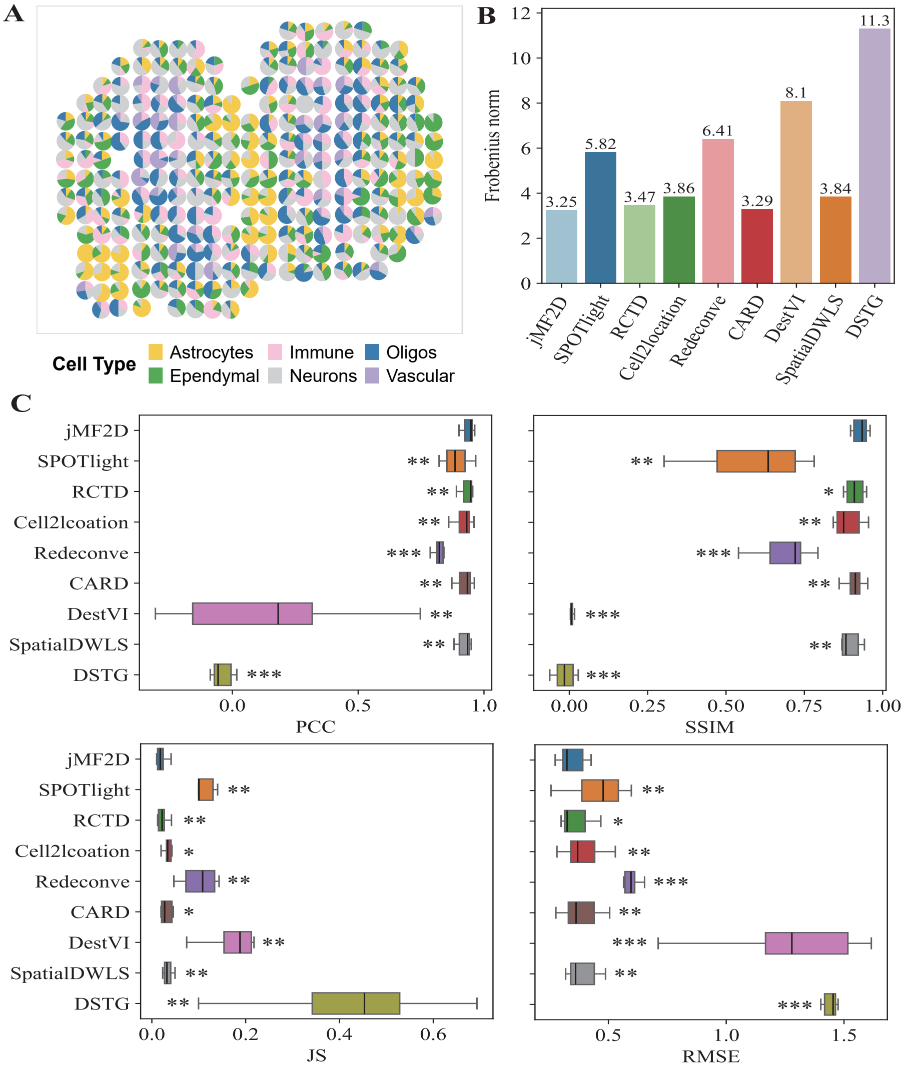

Fig. S4. Performance of various algorithms for cell type deconvolution on simulated spatial transcriptomics data with spatial information. **(A)** Pie chart of the simulated spatial transcriptomics dataset, showing that neighboring spots exhibit similar cell type compositions. **(B)** Distance the predicted and ground truth abundance of cell types in the simulated dataset in terms of Frobenius norm. **(C)** Distribution of performance of various algorithms in terms of PCC, SSIM, JS, and RMSE, where significance is obtained with one-sided student's t-test, and */**/*** denotes Benjamini-Hochberg (FDR-BH) adjusted p-value is less than 5.0E-1/5.0E-2/5.0E-3, respectively.

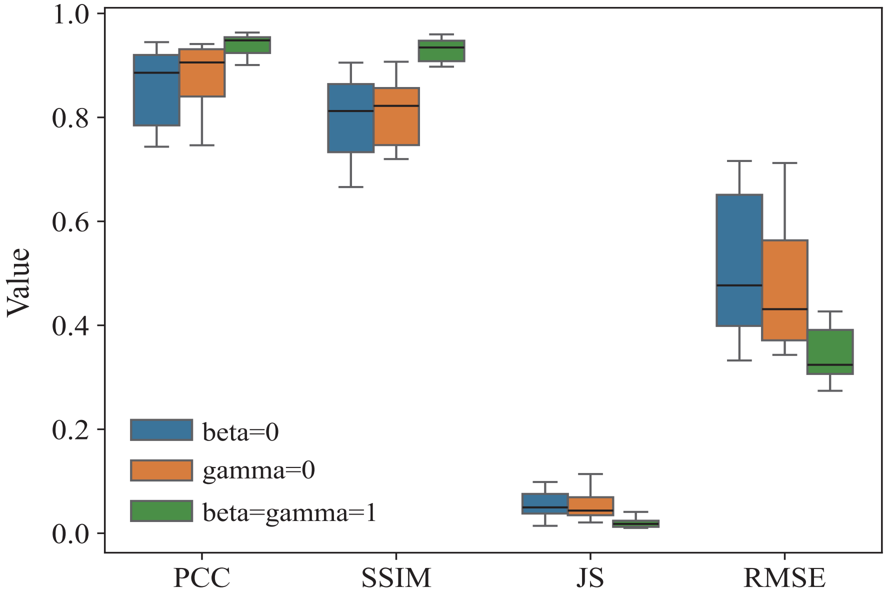

Fig. S5. Ablation study of jMF2D on simulated dataset with spatial information in terms of various measurements.

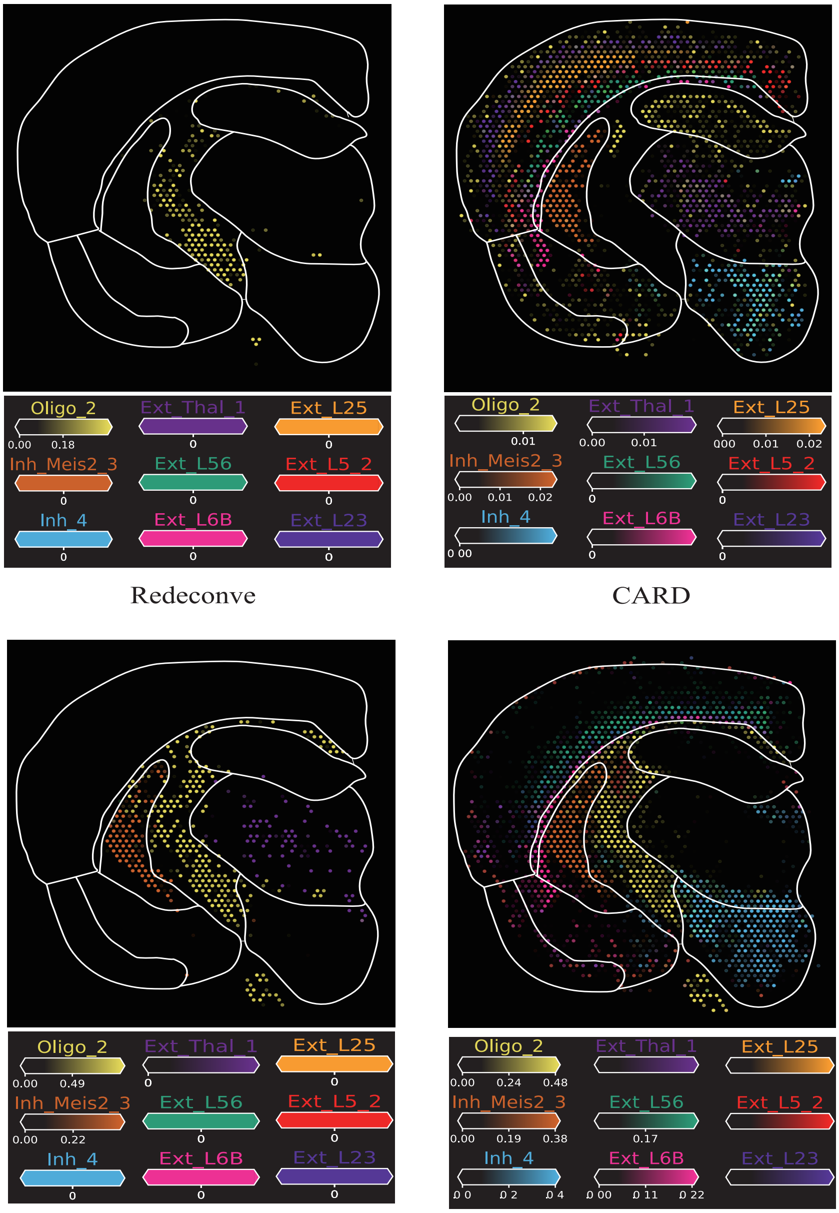

Redeconve CARD

SpatialDWLS DestVI

Fig. S6. Region-specific cell types in the mouse brain obtained by various algorithms, where regions are enclosed by white lines.

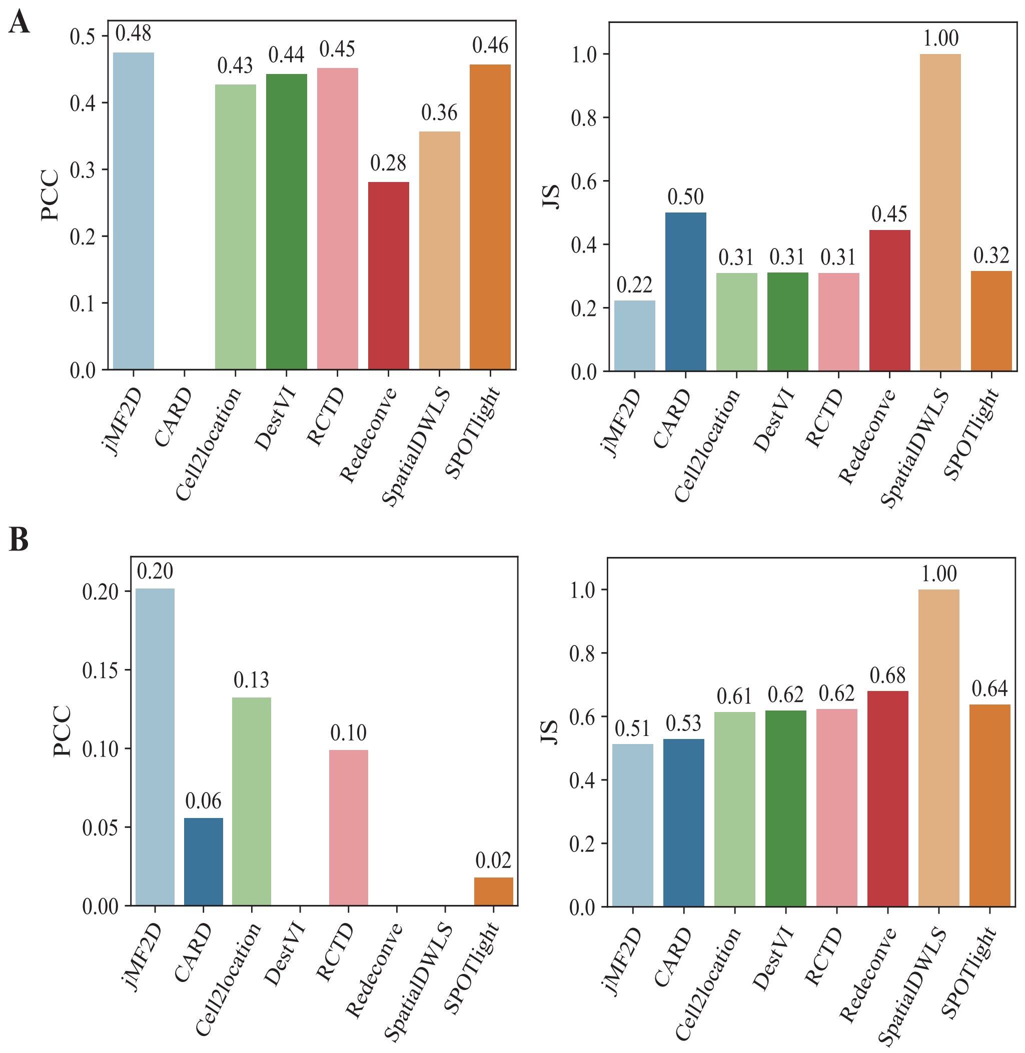

Fig. S7. Performance of various algorithms for mouse brain experiments: **(A)** PCC and JS of various algorithms for deciphering abundance of Oligo_2 with marker gene Prr5l, **(B)** PCC and JS various algorithms for deciphering abundance of Ext_L5_2 with marker gene Gm28928.

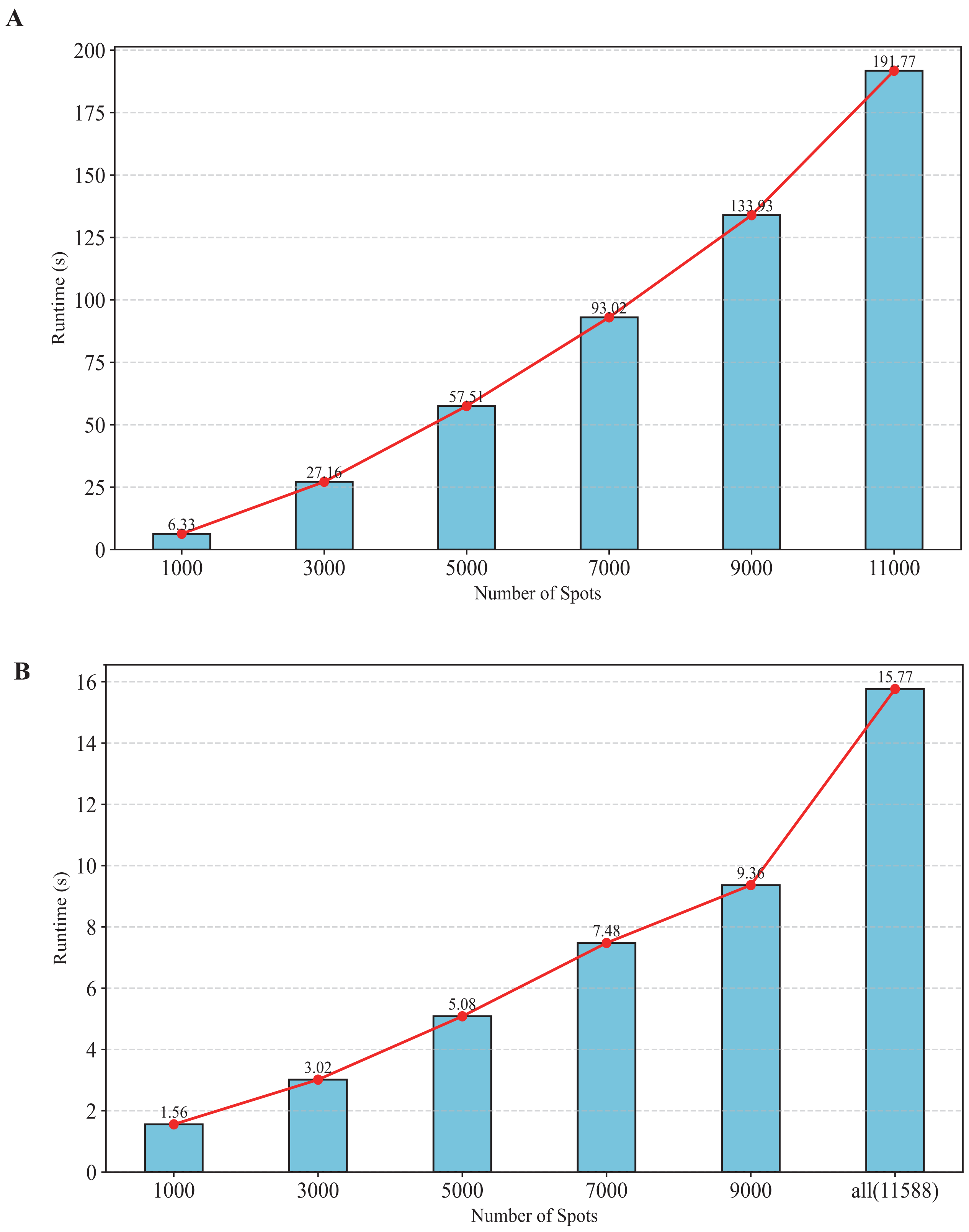

Fig. S8. Scalability analysis of jMF2D with two additional large-scale datasets: (**A**) simulated dataset whose spot number ranging from 1,000 to 11,000 spots, and (**B**) mouse cerebellum dataset with spot number from 1,000 to 11,588 spots, respectively.

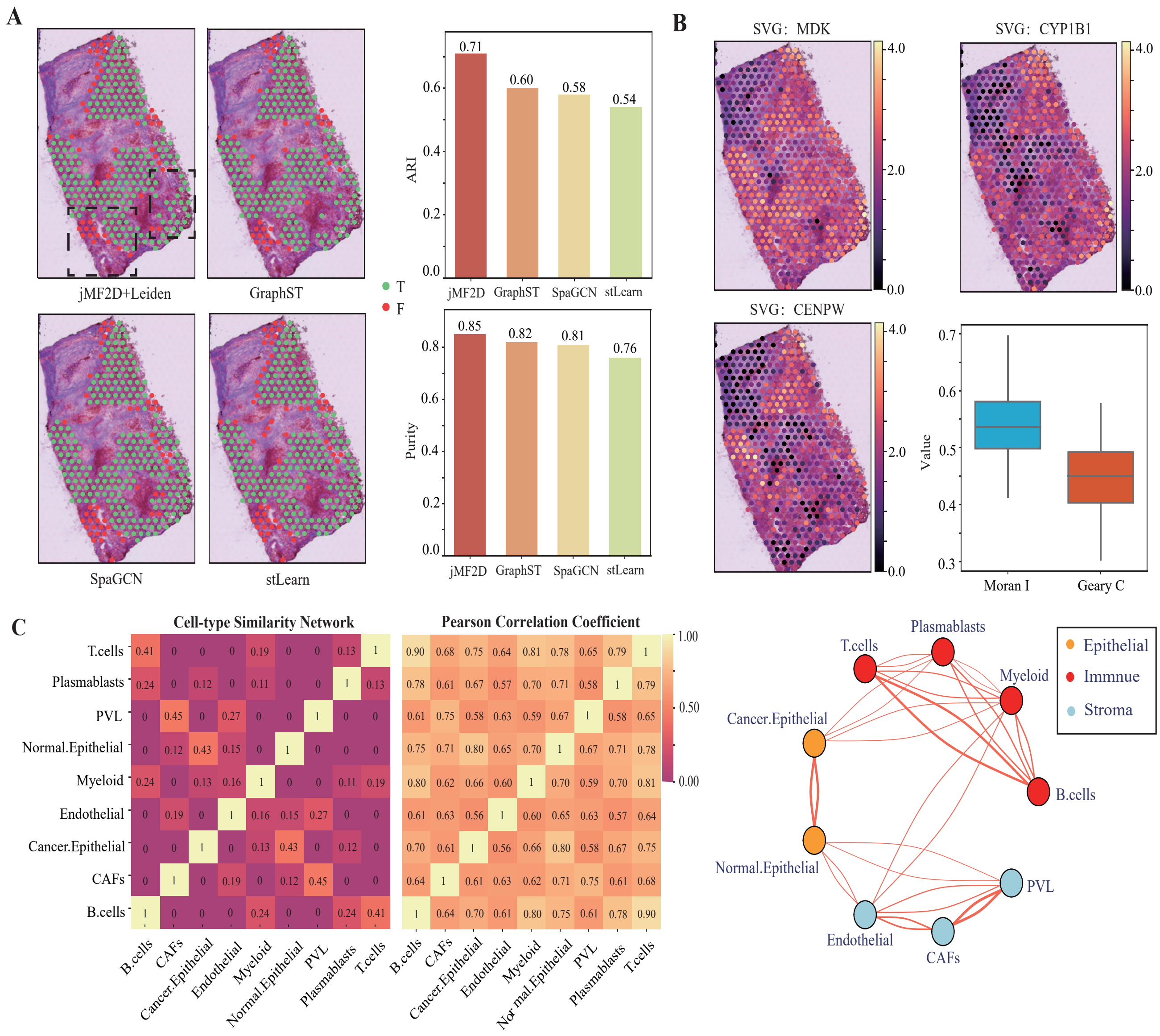

Fig. S9. jMF2D facilitates the identification of cancer-related spatial domains and bio-marker genes from cancer spatial transcriptomics data. **(A)** Left panel visualizes spatial domain of DCIS identified by various algorithms, including jMF2D+Leiden, GraphST, SpaGCN and stLearn, where green and red dots represent the correctly and mistakenly classified spots. Right panel depicts ARI and purity of spatial domain identified by various algorithms. **(B)** Visualization of spatial distribution and expression of top 3 SVGs for spatial domain identified by jMF2D, where expression values are log-transformed. The Moran's I and Geary's C values of the top 100 SVGs from spatial domain identified by jMF2D. **(C)** Heatmap of similarity networks of cell types learned by jMF2D (left) and by using Pearson Correlation Coefficient (middle). Visualization of topological structure of cell type similarity network learned by jMF2D, where nodes are for cell types, edges for relations among nodes, thickness of edges is proportional to edge weights, and colors of nodes denote groups of cell types, respectively.

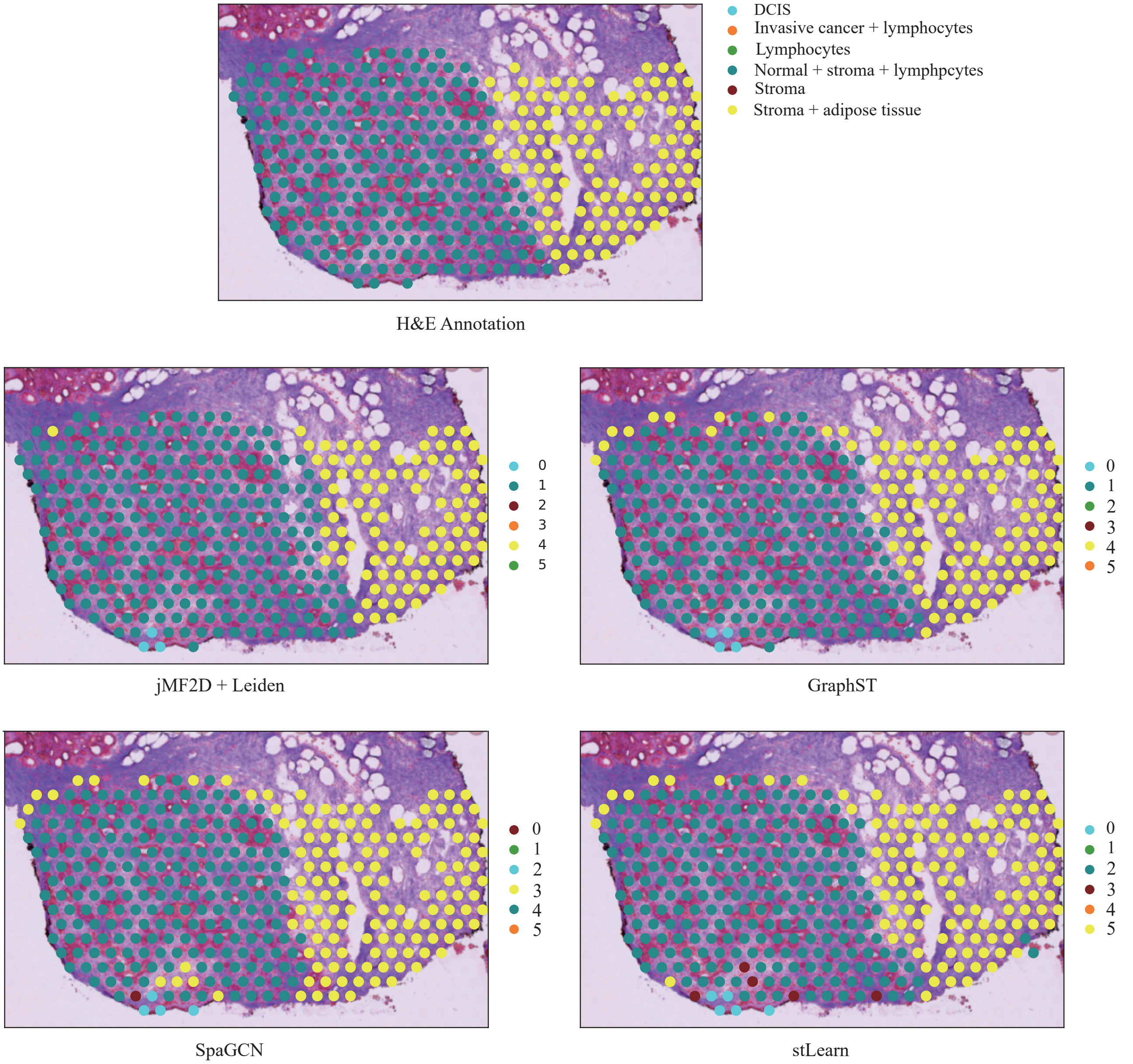

Fig. S10. Spatial domains identified by different algorithms. (Top) Regions annotated as Normal+stroma+lymphocytes and Stroma+adipose tissue by H&E image, and (Bottom) Spatial domains identified by different algorithms, where jMF2D+Leiden represents the use of the jMF2D algorithm for deconvolution followed by clustering with the Leiden algorithm.

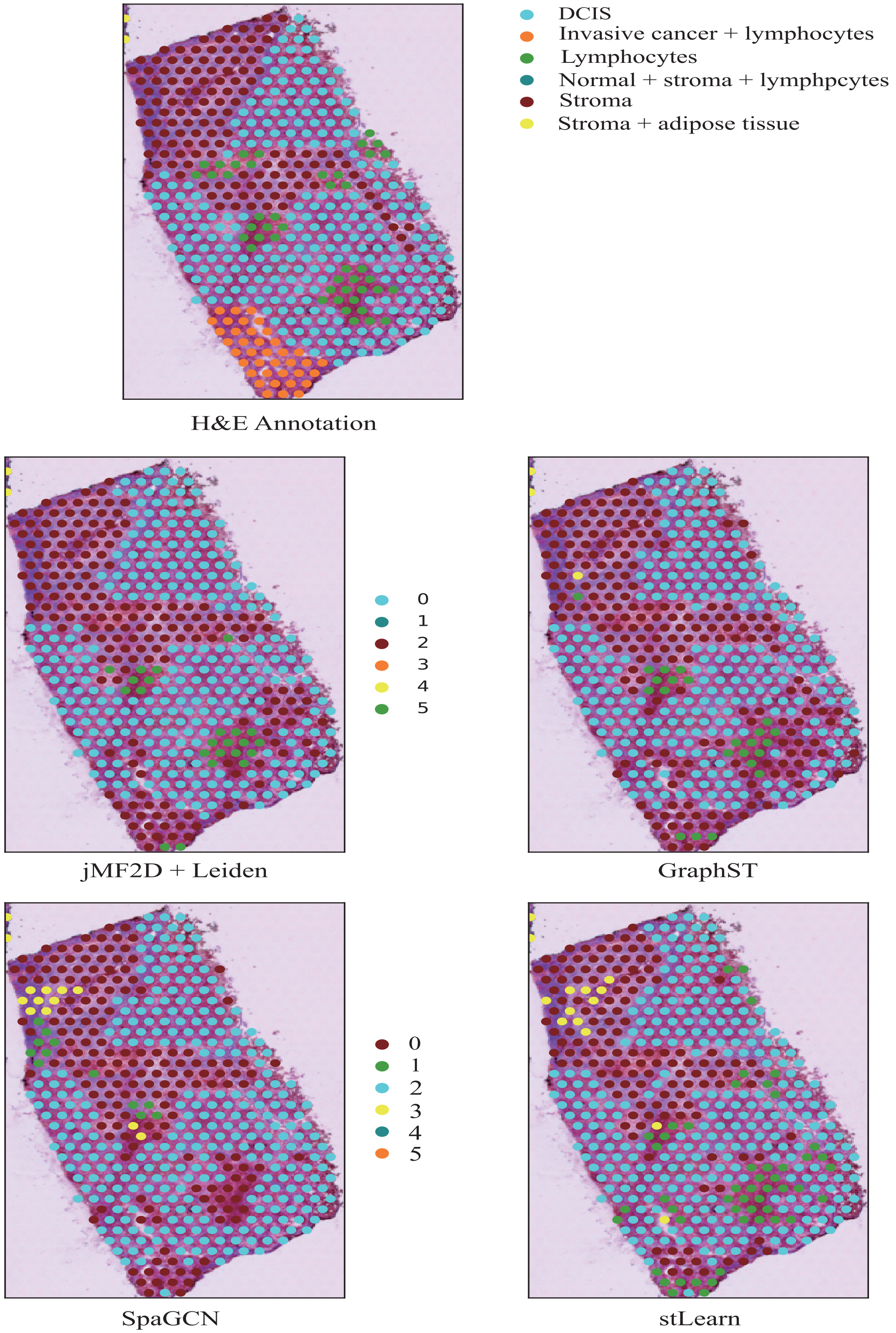

Fig. S11. Spatial domain identified by various algorithms. (Top) Regions annotated as DCIS, Lymphocytes, Stroma and Invasive cancer+lymphocytes by H&E image, and (Bottom) Performance of different algorithms for spatial domain identification, where jMF2D+Leiden represents the use of the jMF2D algorithm for deconvolution followed by clustering with the Leiden algorithm.

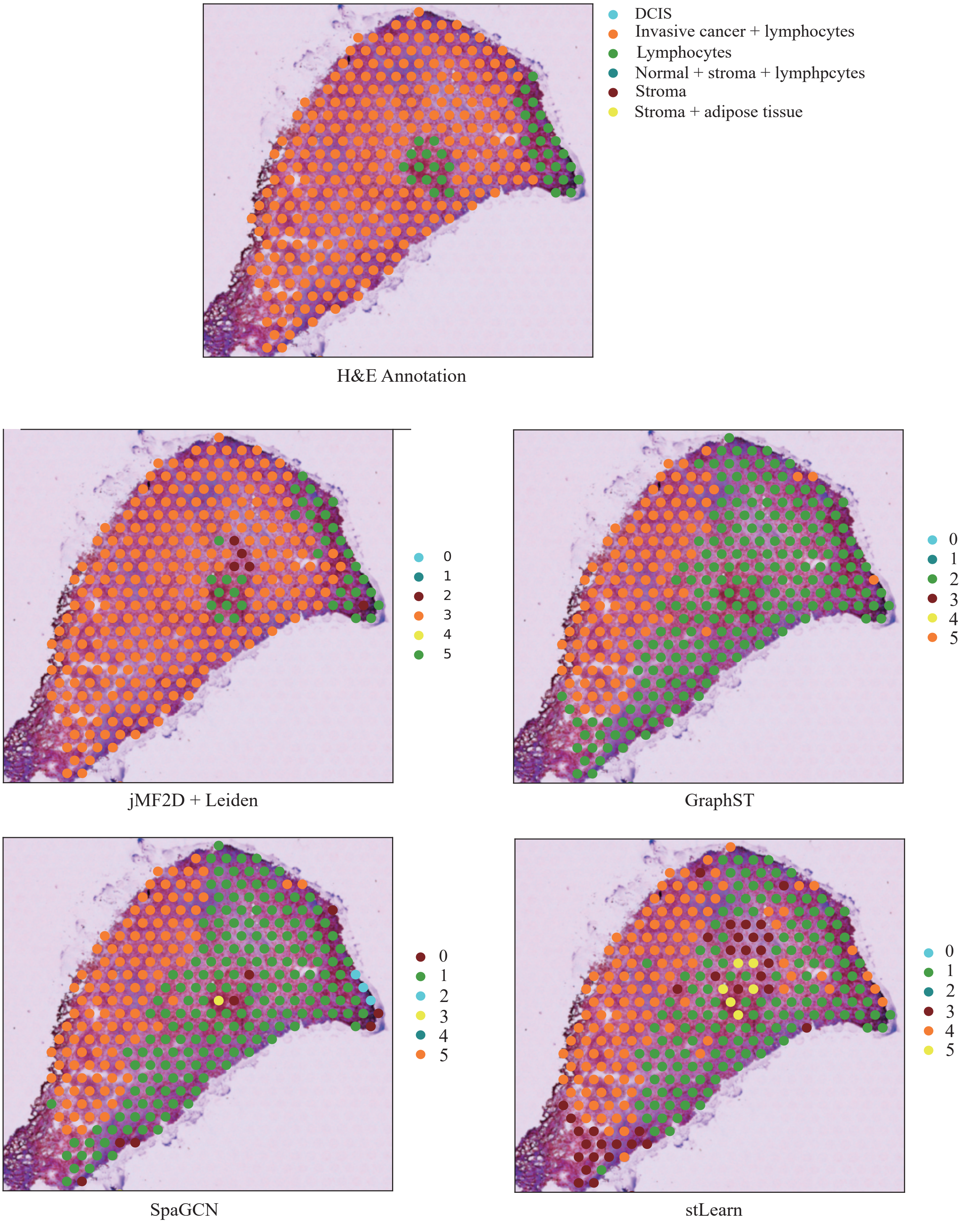

Fig. S12. Spatial domain identified by various algorithms. (Top) Regions annotated as Invasive cancer+lymphocytes and Lymphocytes and by H&E image, and (Bottom) Results of different algorithms for spatial domains, where jMF2D+Leiden represents the use of the jMF2D algorithm for deconvolution followed by clustering with the Leiden algorithm.

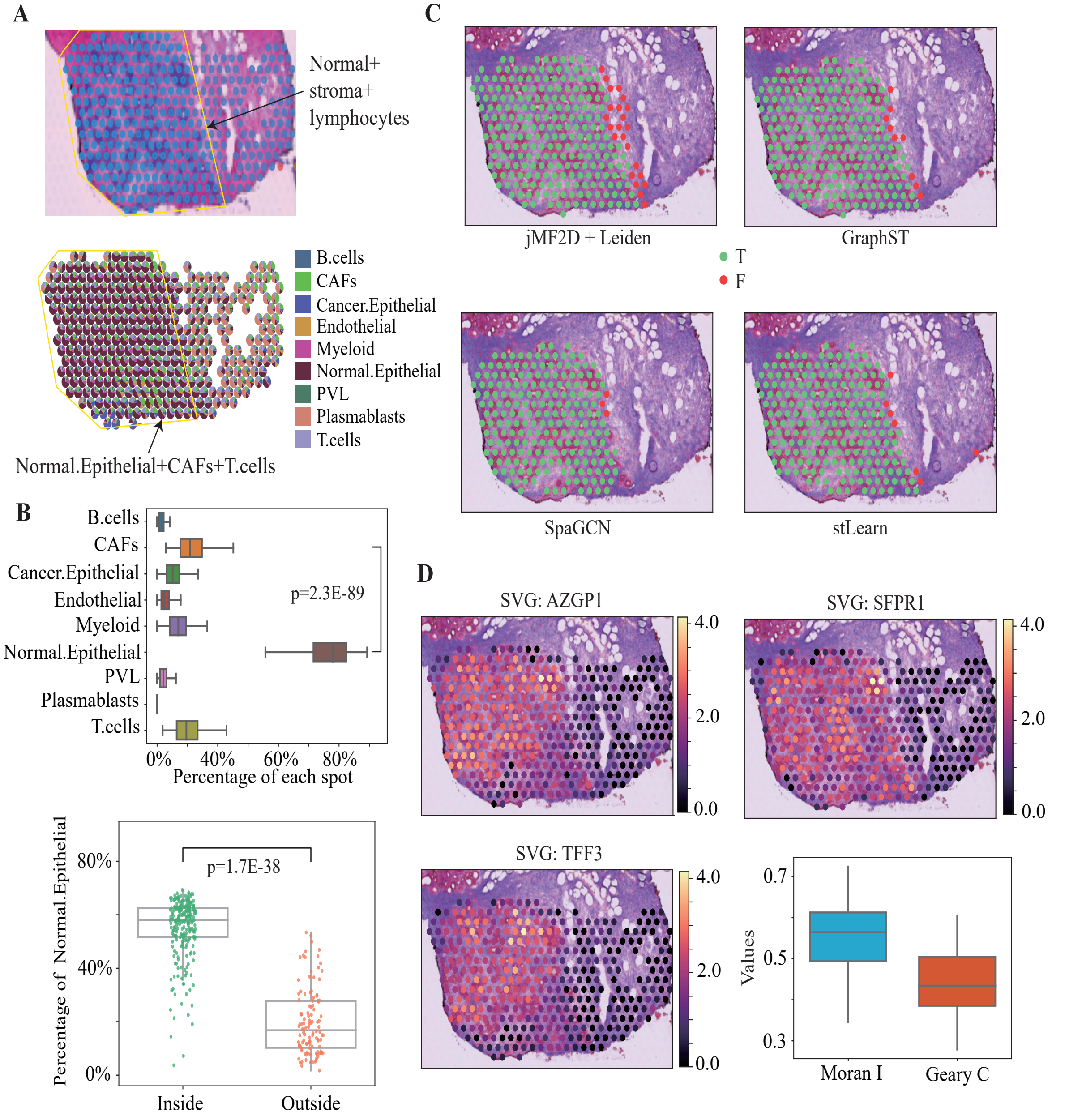

Fig. S13. The results of jMF2D in the normal region of the human breast cancer data. (**A**) The H&E image of normal region of breast cancer that is delineated by solid yellow lines (Top), and spatial scatter pie plot, where the proportions of cell types for each spot are predicted by jMF2D (Bottom). (**B**) The proportions of cell types for each spot within the normal region identified by jMF2D, where Y-axis represents cell types and X-axis denotes proportions of cell types (significance is obtained using a one-sided student's t-test). The bottom panel is distribution of percentage of normal epithelial cells in spots inside and outside of Normal region. (**C**) Visualization of spatial domain of normal region identified by various algorithms, including jMF2D+Leiden, GraphST, SpaGCN and stLearn, where green and red dots represent the correctly and mistakenly classified spots. (**D**) Visualization of spatial distribution of top 3 SVGs. The Moran's I and Geary's C values of the top 100 SVGs from spatial domain identified by jMF2D.

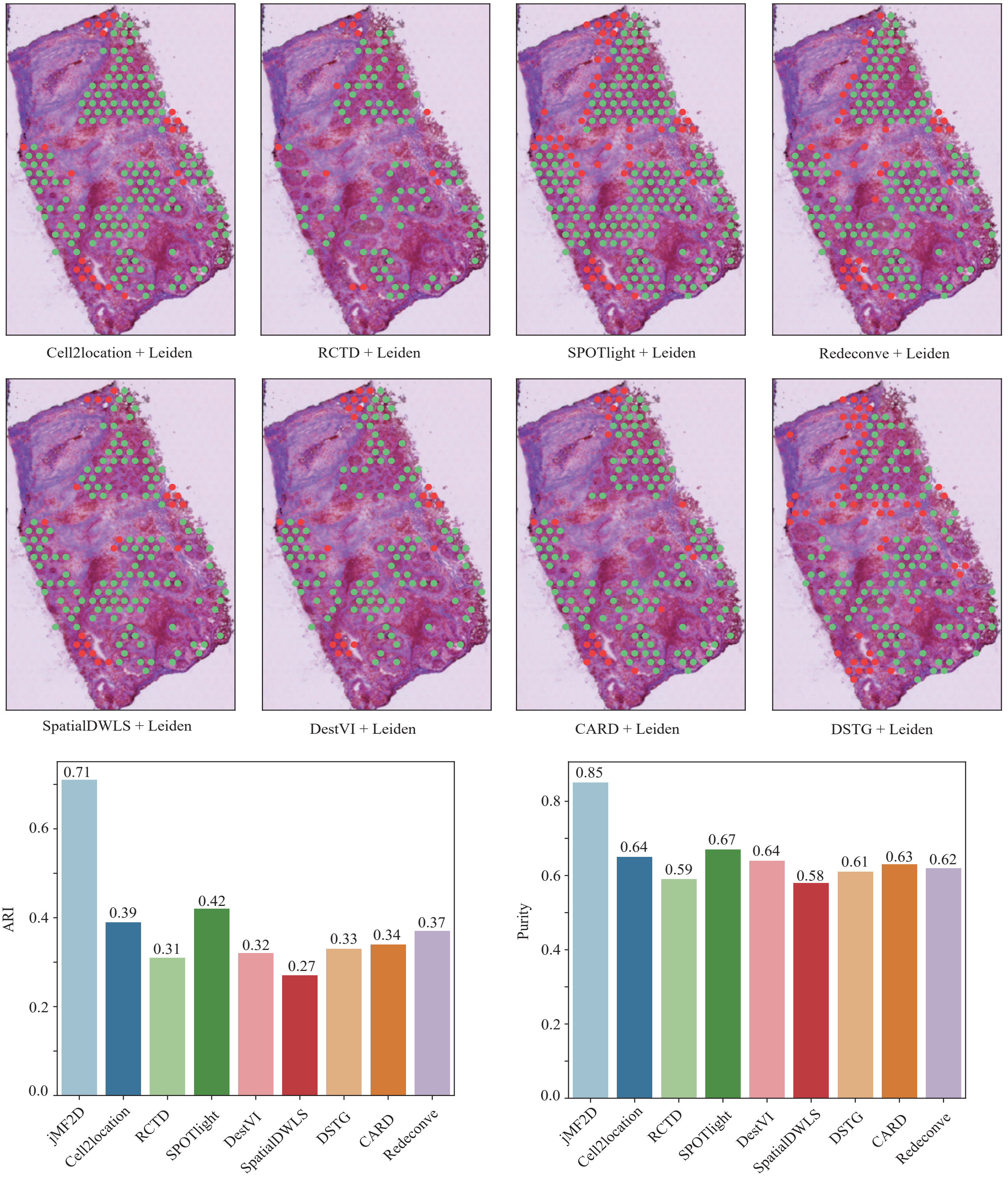

Fig. S14. The results of various algorithms combined with Leiden clustering, where green dots represent spots consistent with the DCIS region in the H&E image, and red dots represent misclassified spots. The ARI and Purity quantify the accuracy of different methods (bottom).

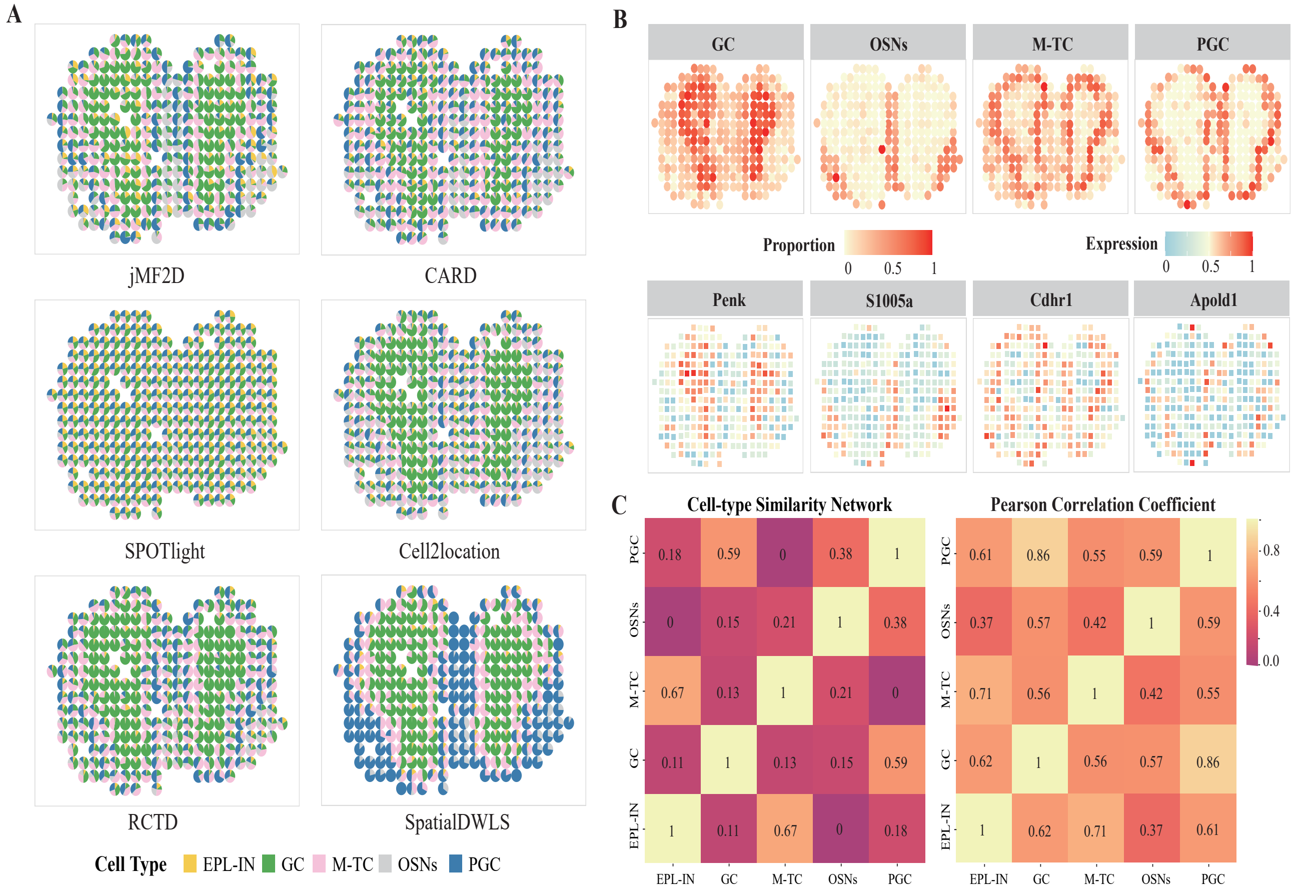

	EPL-IN	GC	M-TC	OSNs	PGC
PGC	0.18	0.59	0	0.38	1
OSNs	0	0.15	0.21	1	0.38
M-TC	0.67	0.13	1	0.21	0
GC	0.11	1	0.13	0.15	0.59
EPL-IN	1	0.11	0.67	0	0.18

	EPL-IN	GC	M-TC	OSNs	PGC
PGC	0.61	0.86	0.55	0.59	1
OSNs	0.37	0.57	0.42	1	0.59
M-TC	0.71	0.56	1	0.42	0.55
GC	0.62	1	0.56	0.57	0.86
EPL-IN	1	0.62	0.71	0.37	0.61

Fig. S15. jMF2D is applicable for spatial transcriptomics data from the mouse olfactory bulb(MOB)generated by Legacy ST platform. (**A**) Spatial scatter pie plot for cell-type composition of each spot of various algorithms. (**B**) Visualization of spatial expression patterns of cell type bio-marker genes for each cell type. (**C**) Heatmaps of cell type similarity networks learned by jMF2D (left) and by calculating Pearson Correlation Coefficient among cell types (right).

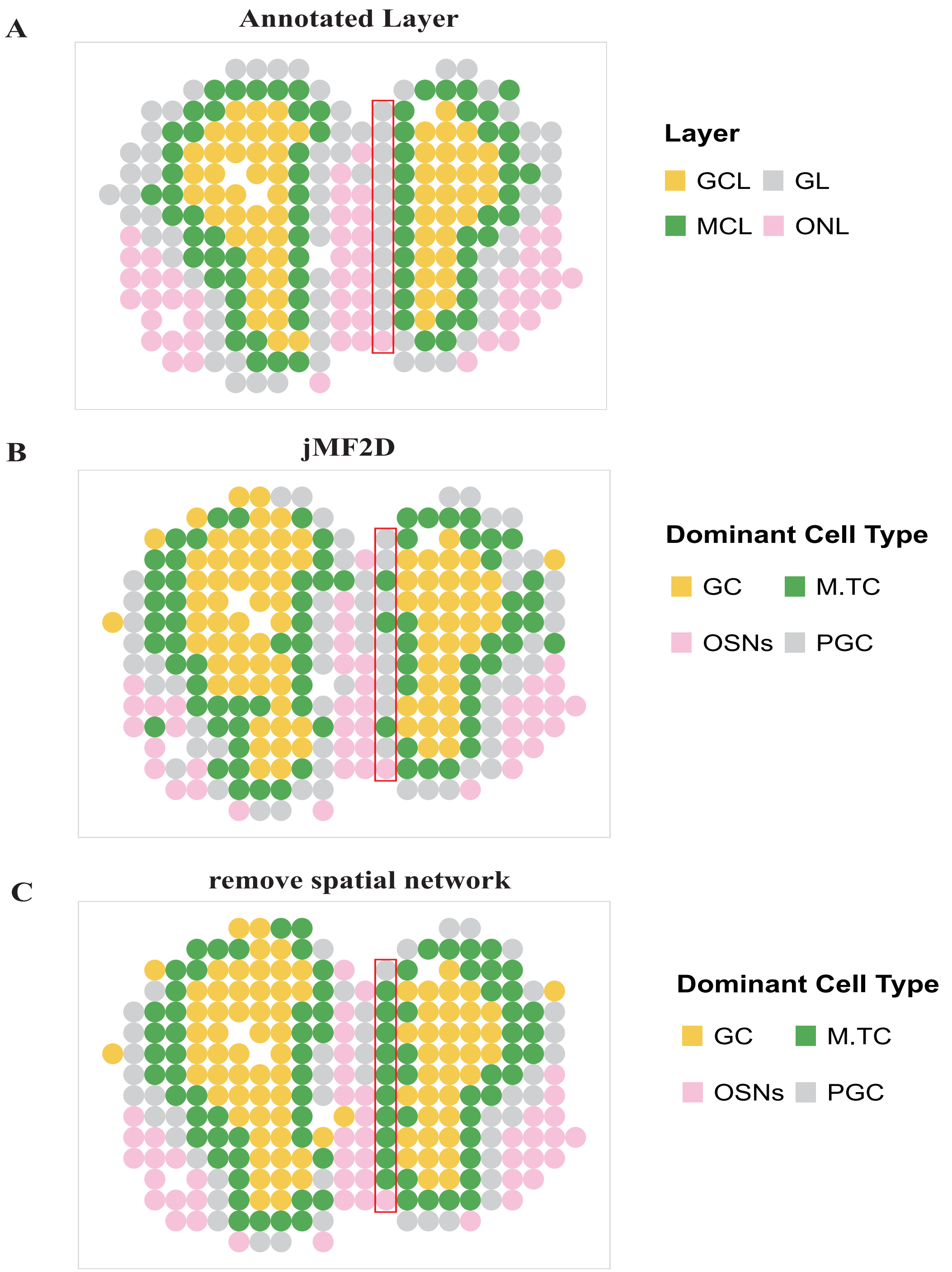

Fig. S16. The layer annotation and the ablation analysis of spatial network. (**A**) H&E staining of the olfactory bulb data with the four anatomical layers. (**B**) The most predominant cell type in each spot identified by jMF2D. (**C**) The dominant cell type identified by jMF2D after removing the spatial network.
